# Antimicrobial biosynthetic potential and diversity of culturable soil actinobacteria from forest ecosystems of Northeast India

**DOI:** 10.1038/s41598-020-60968-6

**Published:** 2020-03-05

**Authors:** Priyanka Sharma, Debajit Thakur

**Affiliations:** 1 Malaria Drug Discovery Laboratory, International Centre for Genetic Engineering and Biotechnology (ICGEB), Aruna Asaf Ali Marg, New Delhi, 110067 India; 2 Microbial Biotechnology Laboratory, Life Sciences Division, Institute of Advanced Study in Science and Technology (IASST), An Autonomous Institute under Department of Science and Technology (Govt. of India), Paschim Boragaon, Garchuk, Guwahati, 781035 Assam India

**Keywords:** Drug discovery, Ecology, Microbiology

## Abstract

Actinobacteria is a goldmine for the discovery of abundant secondary metabolites with diverse biological activities. This study explores antimicrobial biosynthetic potential and diversity of actinobacteria from Pobitora Wildlife Sanctuary and Kaziranga National Park of Assam, India, lying in the Indo-Burma mega-biodiversity hotspot. A total of 107 actinobacteria were isolated, of which 77 exhibited significant antagonistic activity. 24 isolates tested positive for at least one of the polyketide synthase type I, polyketide synthase type II or non-ribosomal peptide synthase genes within their genome. Their secondary metabolite pathway products were predicted to be involved in the production of ansamycin, benzoisochromanequinone, streptogramin using DoBISCUIT database. Molecular identification indicated that these actinobacteria predominantly belonged to genus *Streptomyces*, followed by *Nocardia* and *Kribbella*. 4 strains, viz. *Streptomyces* sp. PB-79 (GenBank accession no. KU901725; 1313 bp), *Streptomyces* sp. Kz-28 (GenBank accession no. KY000534; 1378 bp), *Streptomyces* sp. Kz-32 (GenBank accession no. KY000536; 1377 bp) and *Streptomyces* sp. Kz-67 (GenBank accession no. KY000540; 1383 bp) showed ~89.5% similarity to the nearest type strain in EzTaxon database and may be considered novel. *Streptomyces* sp. Kz-24 (GenBank accession no. KY000533; 1367 bp) showed only 96.2% sequence similarity to *S. malaysiensis* and exhibited minimum inhibitory concentration of 0.024 µg/mL against methicilin resistant *Staphylococcus aureus* ATCC 43300 and *Candida albicans* MTCC 227. This study establishes that actinobacteria isolated from the poorly explored Indo-Burma mega-biodiversity hotspot may be an extremely rich reservoir for production of biologically active compounds for human welfare.

## Introduction

Since ancient times, mankind has been exploring nature for bioactive organisms to treat different ailments. More than 100000 natural products have been identified in the last 150 years, which include highly assorted chemical classes such as polyketides, alkaloids, non-ribosomal peptides, isoprenoids or phenylpropanoids^1,2^. Microbial biodiversity can provide us with the richest and the most versatile reservoir of potentially active natural products. A large portion of genomes from beneficial microbes are dedicated to the production of these valuable natural products. A single microbe is capable of making 30–50 natural product compounds^3^. There are approximately 1 million natural products out of which about 25% are biologically active showing either positive activity or toxicity. Among the prospective sources of natural products, bacteria are prolific sources and a vast majority of these products are produced by a phylum of Gram-positive bacteria known as the “Actinobacteria”^4–6^.

Actinobacteria are diverse group of bacteria with high content of guanosine-cytosine (65–75%) ranging from 2.5–9.7 Mb genome size^7^. Among actinobacteria, about 75% of antibiotics such as ivermectin, streptomycin, nystatin and tetracycline are produced by the microbial world’s richest antibiotic-producing family known as “Streptomycetes” ^8,9^. Drug resistance has been found in many bacterial pathogens due to the misuse or overuse of antibiotics over a prolonged period. The number of fungal infections is also increasing. The economic burden associated with these infections is immense and pharmaceutical industries have been reluctant to invest in antibiotic research and development^10,11^. For the development of novel pharmaceuticals, new biologically active compounds have to be found. This necessity has led to the search for new bioactive compounds among poorly explored habitats which can efficiently target these life-threatening pathogens^12^. Hence, actinobacteria may be a potential solution to these problems. In order to find new microbial products derived from newly identified activities of actinobacteria, the search has to be shifted from routinely explored ecological niches to unexplored ones^13,14^.

Northeast India, a part of the Indo-Burma mega-biodiversity hotspot, is well known for its rich biodiversity. The region has diverse climatic, edaphic and altitudinal variations resulting in wide ecological habitats. Northeast India is the connection between the Indian, Indo-Malayan and Indo-Chinese bio-geographic regions. It is home to a wide spectrum of India’s flora and fauna^15^. Unlike the floral and faunal diversity, the microbial diversity is also relatively unexplored in this part of the world. The local environment may influence the evolution of novel secondary metabolic pathways in organisms found in the biodiversity hotspots^16^. Forests are considered to be the most bio-diversified terrestrial ecosystems on earth. They are the largest possible resource available to obtain novel microorganisms and their valuable natural products^17,18^. Quite a few researchers have reported actinobacteria from the forest ecosystems of Northeast India for the search of natural products endowed with antimicrobial activity^19–26^. However, the literature review reveals that Pobitora Wildlife Sanctuary, located in Assam, India is yet an unexplored forest ecosystem and we had previously reported regarding the bioactivity prospective of its microflora^24^. Thus, this forest ecosystem can be considered as an unexplored source of actinobacteria producing bioactive metabolites. Regarding the study of actinobacteria from Kaziranga National Park of Assam, this forest has been previously explored by few researchers and as such, holds a lot of promise for isolation of actinobacteria having pharmaceutical potential^19,20^.

In the light of the above studies, the present investigation was undertaken with an aim to isolate actinobacteria from Pobitora Wildlife Sanctuary (26 °12′ to 26 °16′N and 91 °58′ to 92 °05′E) and Kaziranga National Park (26 °30′ to 26 °45′N; 93 °08′ to 93 °36′E) of Assam, India and screening them against an array of microbial pathogens responsible for human pathogenesis. Emphasis was given for screening of the isolates for the presence of antibiotic biosynthetic genes and the different chemical classes of antimicrobial compounds they produce were also predicted using DoBISCUIT. Analysis of genetic diversity of the actinobacteria isolates was carried out by 16S rDNA-ARDRA. An attempt was made to study a promising actinobacteria exhibiting potent antimicrobial potential against a wide range of microorganisms.

## Results

### Isolation of actinobacteria

A total of 107 presumptive actinobacteria of different phenotypes were isolated from different environmental sites of Pobitora Wildlife Sanctuary (n = 54) and Kaziranga National Park (n = 53) of Assam, India. The details of the nature of soil samples, its pH and the number of actinobacteria isolated are given in Table 1. They were associated with scanty to profuse sporulating capacity and showed the presence of distinctive colonial morphology, mycelia colour and pigment production (See Supplementary Table S1). The white colour series was found to be the most dominant one (n = 48; 44.8%). The light microscopy results showed the spiral chain morphology of the aerial mycelia (See Supplementary Fig. S1).

**Table 1:** Description of soil samples and number of actinobacteria isolated from each sample.

Sample No.	Type of soil sample	Soil pH	No. of actinobacteria
**Pobitora Wildlife Sanctuary, Assam, India**			
Sample 1	Grass rhizosphere soil	5.7	13
Sample 2	Leaf litter soil	4.6	8
Sample 3	Tree rhizosphere soil	4.9	17
Sample 4	Sediment soil	4.5	16
**Kaziranga National Park, Assam, India**			
Sample A	Leaf litter soil	5.3	10
Sample B	Tree rhizosphere soil	4.5	9
Sample C	Sediment soil	5.6	10
Sample D	Tree rhizosphere soil	6.0	8
Sample E	Leaf litter soil	5.6	16
Total presumptive actinobacteria			107

### Antimicrobial assay of actinobacteria

During preliminary screening, all the 107 actinobacteria were assessed against four test microorganisms for their potential to produce antimicrobials. 77 actinobacteria (71.9%) exhibited positive activity out of which 39 isolates are from Pobitora Wildlife Sanctuary and 38 isolates are from Kaziranga National Park. A total of 51 isolates (66%) showed antimicrobial activity against *Staphylococcus aureus* MTCC 96 with maximum zone of inhibition (70 ± 1.3) mm by Kz-32. 49 isolates (64%) exhibited antimicrobial activity against methicilin resistant *Staphylococcus aureus* (MRSA) ATCC 43300 with maximum zone of inhibition of (56 ± 1) mm by Kz-24. Against *Escherichia coli* MTCC 40, 59 isolates (77%) showed antimicrobial activity with highest zone of inhibition of (56 ± 0.8) mm diameter by PB-65. 60 isolates (78%) exhibited antimicrobial activity against *Candida albicans* MTCC 227 where highest inhibition zone was observed by Kz-24 with (52 ± 1.8) mm. Furthermore, 29 isolates (37.6%) showed antimicrobial activity against all the four test microorganisms. Results of antimicrobial activity screening of actinobacteria by spot inoculation method are shown in Table 2.

**Table 2:** *In vitro* antimicrobial activity of actinobacteria isolated from forest ecosystems of Assam, India by spot inoculation method.

Sl. No.	Isolate code	*Zone of inhibition (mm)			
		Bacterial test microorganisms			Fungal test microorganism
		*S. aureus* MTCC 96	MRSA ATCC 43300	*E. coli* MTCC 40	*C. albicans* MTCC 227
1.	PB-9	11^a^ ± 0.5	ND	12^ab^ ± 1.2	ND
2.	PB-10	ND	ND	ND	13^abcdef^ ± 1
3.	PB-15	26^l^ ± 1	14^efgh^ ± 2	18^hi^ ± 1.5	20^n^°^p^ ± 1
4.	PB-17	31^mn^ ± 1	ND	ND	11^a^ ± 1
5.	PB-19	14 ^cdefg^ ± 1.5	11^ab^ ± 1.6	ND	13^abcdef^ ± 2
6.	PB-20	ND	ND	12^ab^ ± 0.7	ND
7.	PB-21	32^n^ ± 0.7	ND	55^x^ ± 1.5	20^nop^ ± 1
8.	PB-22	ND	ND	ND	15^efghij^ ± 1.5
9.	PB-25	20^ij^ ± 1.2	11^abc^ ± 1.7	ND	15^cdefghi^ ± 1.5
10.	PB-26	13^abc^ ± 1	ND	15^ef^ ± 1.7	ND
11.	PB-27	ND	ND	30^tu^ ± 2	ND
12.	PB-28	19^hi^ ± 2	20° ± 0.7	35^v^ ± 0.5	18^lmn^ ± 2.7
13.	PB-31	ND	ND	46^w^ ± 3	ND
14.	PB-32	ND	ND	20^ij^ ± 0.7	12^abc^ ± 1
15.	PB-33	18^h^ ± 0.7	17^jkl^ ± 1.3	20^ij^ ± 1.2	12^abcd^ ± 1.2
16.	PB-39	14^cde^±1	12^abcd^ ± 2	28^qrs^ ± 0.5	14^bcdefgh^ ± 1.7
17.	PB-43	26^l^ ± 1.5	18^lmn^ ± 1	27^pqrs^ ± 2	12^ab^ ± 0.7
18.	PB-46	ND	ND	ND	36^x^ ± 1
19.	PB-47	14^bcd^ ± 1.5	ND	ND	16^ghijkl^ ± 1.5
20.	PB-48	15^defg^ ± 1	19^no^ ± 1.2	30^tu^ ± 1.5	40^z^ ± 1.7
21.	PB-50	38^p^ ± 0.7	ND	15^ef^ ± 1	ND
22.	PB-51	14^cdef^ ± 1.2	15^ghij^ ± 2	28^rs^ ± 1	17^hijklm^ ± 1.4
23.	PB-52	36^o^ ± 0.8	30^r^ ± 0.8	31 ^u^ ± 1.2	27^u^ ± 0.4
24.	PB-54	15^cdefg^ ± 1.2	12^abcd^ ± 1.6	36 ^v^ ± 1	14^bcdefg^ ± 2
25.	PB-55	ND	ND	13^abc^ ± 1.3	15^fghijk^ ± 1
26.	PB-56	15^cdefg^ ± 2.5	ND	14^cdef^ ± 0.5	18^lmn^ ± 1
27.	PB-64	26^l^ ± 1.2	25^p^ ± 0.5	14^bcde^ ± 0.5	17^ijklm^±1.5
28.	PB-65	41^q^ ± 1	28^q^ ± 0.7	56^x^±0.8	15^fghijk^ ± 1.2
29.	PB-66	ND	ND	ND	37^xy^ ± 1
30.	PB-68	60^s^ ± 0.5	13^def^ ± 1.7	20^ij^ ± 1	18l^mn^ ± 2.7
31.	PB-70	31^n^ ± 1.5	30^r^ ± 1.5	55^x^ ± 0.7	14^bcdef^ ± 2
32.	PB-75	19^hi^ ± 1.7	ND	15^def^ ± 1.5	19^mno^ ± 1.7
33.	PB-76	16^fg^ ± 1	20^o^ ± 1.2	13^abcde^ ± 1.7	13^abcde^ ± 1.3
34.	PB-79	12^ab^ ± 1	ND	25^nop^ ± 2	ND
35.	PB-81	16^efg^ ± 0.7	31^r^ ± 1.5	19^hij^ ± 1.2	ND
36.	PB-82	ND	ND	16^fg^ ± 1	ND
37.	PB-83	14^cdef^ ± 1.2	10^a^ ± 2	27^opqr^ ± 1.5	17^jklm^ ± 1
38.	PB-85	21^jk^ ± 0.7	13^bcde^ ± 1.3	46^w^ ± 1.2	12^abc^ ± 1
39.	PB-86	19^hi^ ± 1	30^r^ ± 1.2	30^tu^ ± 2	22^pqr^ ± 0.8
40.	Kz-2	ND	13^cde^ ± 1	14^cdef^ ± 1.5	15^fghijk^ ± 0.5
41.	Kz-10	ND	17^klm^ ± 1.2	12^a^ ± 1.3	18^lmn^ ± 0.7
42.	Kz-11	ND	15^ghij^	16^fg^ ± 0.7	17^klm^ ± 1
43.	Kz-12	25^l^ ± 1.5	13^defg^ ± 1.5	35^v^ ± 0.7	ND
44.	Kz-13	15^defg^ ± 1	14^efgh^ ± 1	21^jk^ ± 0.7	18^lmn^ ± 2
45.	Kz-14	ND	16^ijkl^ ± 1.2	26^nopq^ ± 0.7	23^rs^ ± 1
46.	Kz-18	ND	16^ijkl^ ± 1.5	31^tu^ ± 0.7	20^nopq^ ± 1.2
47.	Kz-21	ND	19^mno^ ± 0.5	26^nopq^ ± 0.7	21^opqr^ ± 1.5
48.	Kz-23	16^g^ ± 1.2	ND	20^ij^	23^qrs^ ± 0.8
49.	Kz-24	42^q^ ± 2	56^t^ ± 1	26^nopq^ ± 0.5	52^Ψ^ ± 1.8
50.	Kz-27	ND	ND	20^ij^ ± 1	25^tu^ ± 1.2
51.	Kz-28	29^m^ ± 0.5	25^p^ ± 1	24^lmn^ ± 2	31^v^ ± 0.7
52.	Kz-29	31^mn^ ± 0.5	25^p^ ± 1.5	27^opqr^ ± 2	ND
53.	Kz-31	ND	ND	ND	17^jklm^ ± 0.7
54.	Kz-32	70^t^ ± 1.3	12^abcd^ ± 0.7	ND	30^v^ ± 1.5
55.	Kz-36	51^r^ ± 1	ND	29^st^ ± 0.7	34^w^ ± 1
56.	Kz-38	ND	15^ghij^ ± 1	ND	15^defghij^ ± 0.5
57.	Kz-41	31^n^ ± 1.2	16^hijk^ ± 1	20^ij^ ± 0.2	21^opqr^ ± 1
58.	Kz-42	ND	ND	ND	12^ab^ ± 1.7
59.	Kz-44	20^ij^ ± 1	14^efghi^ ± 1.5	20^ij^ ± 0.7	14^cdefghi^ ± 1.5
60.	Kz-47	20^ij^ ± 2	10^a^	20^ij^ ± 1	40^yz^ ± 1.2
61.	Kz-49	15^defg^ ± 1	15^ghij^ ± 1.2	ND	ND
62.	Kz-52	13^abc^ ± 2	25^p^ ± 0.5	ND	15^efghij^ ± 1.5
63.	Kz-55	31^mn^ ± 0.7	16^hijk^ ± 0.8	20^hij^ ± 1	17^ghijkl^ ± 1.5
64.	Kz-56	21^jk^ ± 1	15^hijk^ ± 1.5	25^mno^ ± 0.5	ND
65.	Kz-58	ND	16^hijk^ ± 1	23^lm^ ± 1.7	ND
66.	Kz-61	ND	ND	18^hij^ ± 0.5	ND
67.	Kz-62	31^n^ ± 1.5	37^s^ ± 0.7	ND	ND
68.	Kz-66	19^hi^ ± 1	12^bcd^	19^hij^ ± 2	20^nop^ ± 1.7
69.	Kz-67	22^k^ ± 0.5	14^efgh^ ± 1.7	26^opqr^ ± 1.2	19^mno^ ± 1
70.	Kz-72	ND	ND	ND	21^opqr^ ± 1
71.	Kz-73	ND	ND	ND	22^pqrs^ ± 2
72.	Kz-74	25^l^ ± 1.2	15^fghij^ ± 1.7	23^kl^ ± 0.8	26^u^ ± 1.5
73.	Kz-75	ND	ND	ND	24^st^ ± 1.2
74.	Kz-76	26^l^ ± 0.8	12^bcd^ ± 1	18^gh^ ± 1	ND
75.	Kz-78	25^l^	13^cde^ ± 0.7	18^gh^ ± 1.5	30^v^ ± 2
76.	Kz-79	ND	15^ghij^ ± 1.2	13^abcd^ ± 1	17^jklm^ ± 1.6
77.	Kz-80	21^ij^ ± 1.8	38^s^ ± 1.2	19^hij^ ± 1	20^nop^ ± 2

The average size of colony of the actinobacteria isolates in GLM agar were (7 ± 2) mm in diameter after 5–10 days of incubation at 28 °C.

*Zone of inhibition by spot inoculation method on GLM agar medium.

Zone of inhibition values are given as mean ± SD (n = 3). Values having different superscripts (a–z, Ψ) differ significantly (*P* < 0.05).

ND: not detectable.

Based on the preliminary antimicrobial activity, 19 antagonistic actinobacteria were tested against ten test microorganisms for secondary antimicrobial screening in liquid media. The result of antimicrobial activity of ethyl acetate extracted product of actinobacteria is presented in Supplementary Table S2. From the result, it was observed that all the 19 isolates had the ability to inhibit *S. aureus* MTCC 96, *Micrococcus luteus* MTCC 1538, *E. coli* MTCC 40, *Pseudomonas aeruginosa* MTCC 741 and *C. albicans* MTCC 227. However, 12 isolates, i.e. PB-15, PB-28, PB-43, PB-48, PB-52, PB-64, PB-65, PB-68, PB-76, Kz-13, Kz-55 and Kz-74 had the ability to inhibit all the test microorganisms. 10% DMSO which served as negative control did not show any antimicrobial activity. Antimicrobial activity of the isolates by spot inoculation method and disc diffusion method against test microorganisms is shown in Supplementary Fig. S2.

### Extracellular enzymes production

Out of the 77 antagonistic actinobacteria, 63 (82%) produced amylase, 56 isolates (73%) produced cellulase, 53 isolates (69%) produced protease, 59 isolates (77%) produced lipase and 58 isolates (75%) produced esterase (See Supplementary Table S3). Interestingly, 24 isolates (31%) produced all the five enzymes tested. The detailed data of enzymes production by the isolates is represented by Venn diagram in Supplementary Fig. S3.

### Detection and analysis of PKS-I, PKS-II and NRPS genes for prediction of chemical classes

All the 77 antagonistic actinobacteria were evaluated for their biosynthetic potential in terms of natural product drug discovery. 24 isolates indicated the presence of at least one of the PKS-I, PKS-II or NRPS genes. PKS-I genes were detected in 6 isolates, PKS-II in 20 isolates and NRPS genes were detected in 2 isolates. The partial gene sequences of PKS-I, PKS-II and NRPS were deposited in GenBank under the following accession numbers KY073865-KY073869, KY235144-KY235162, KU721842, KU721843, KY271082 and KY274457 (Table 3).

**Table 3:** Amino acid sequence similarities of the PKS-I, PKS-II and NRPS genes of the actinobacteria and predicted chemical classes for functional genes.

Isolate	GenBank accession no.	Top blast match (GenBank accession no)	Similarity (%)	*Predicted pathway product (by DoBISCUIT)	Product classification	Activity reported
**PKS-I**						
PB-10	KY073865	Modular polyketide synthase of *S. neyagawaensis* ATCC 27449 (AAZ94386)	55	Concanamycin A	Macrocyclic lactone	Antifungal, Antiprotozoal, Antitumor, Antiviral^57^
PB-32	KY073866	Type I modular polyketide synthase of *S. spiroverticillatus* (ABW96540)	55	Tautomycin	Tetronic Acid Derivative	Antibacterial, Antifungal, Antitumor^58^
PB-47	KY073867	ChlA1 polyketide synthase of *S.antibioticus* DSM 40725 (AAZ77693)	58	Chlorothricin	Tetronic acid derivative	Antibacterial^119^
PB-52	KU721843	NanA8 polyketide synthase of *S. nanchangensis* NS3226 (AAP42874)	56	Nanchangmycin	Polyether	Antibacterial, Insecticidal^120^, Ionophore^121^
PB-64	KY073868	Modular polyketide synthase of *S. avermitilis* ATCC 31267 (BAB69192)	58	Oligomycin	Macrocyclic lactone	Antifungal, Antitumor^27^
Kz-24	KY073869	RifA polyketide synthase of *Amycolatopsis mediterranei* S699 (AAC01710)	68	Rifamycin	Ansamycin	Antibacterial^122^
**PKS-II**						
PB-9	KY235144	β-ketoacyl synthase of *S. echinatus* Tu303 (ABL09959)	71	Aranciamycin	Anthracycline	Antibacterial, Collagenase inhibitor^123^
PB-10	KY271082	Ketoacyl synthase of *S. violaceoruber* Tu22 (CAA09653)	81	Granaticin	Benzoisochromanequinone	Antibacterial^124^
PB-15	KY235145	Putative ketoacyl synthase of *S. fradiae* Tu2717 (CAA60569)	74	Urdamycin	Angucycline	Antibacterial, Antitumor^59^
PB-22	KY235146	β-ketoacyl synthase of *S. arenae* DSM 40737 (AAD20267)	72	Naphthocyclinone	Naphthoquinone, Isochromanequinone	Antibacterial^125^
PB-33	KY235147	β-ketoacyl synthase of *S. coelicolor* A3(2) (CAA45043)	73	Actinorhodin	Benzoisochromanequinone	Antibacterial^126^
PB-47	KY235148	Ketoacyl synthase of *S. argillaceus* ATCC 12956 (CAA61989)	78	Mithramycin	Aureolic acid	Antibacterial, Antitumor^127^
PB-48	KY235149	Jadomycin polyketide ketosynthase of *S. venezuelae* ATCC 10712 (AAB36562)	72	Jadomycin B	Angucycline	Antibacterial^128^
PB-64	KY235150	Jadomycin polyketide ketosynthase of *S. venezuelae* ATCC 10712 (AAB36562)	94	Jadomycin B	Angucycline	Antibacterial^128^
PB-65	KY235151	Putative ketoacyl synthase of *Streptomyces* sp. SCC-2136 (CAH10117)	89	Sch 47554	Angucycline	Antifungal^8^
PB-66	KY235152	Putative ketoacyl synthase of *S. fradiae* Tu2717 (CAA60569)	95	Urdamycin	Angucycline, Benzanthraquinone	Antibacterial, Antitumor^59^
PB-68	KY274457	β-ketoacyl synthase of *Streptomyces* sp. AM-7161 (BAC79045)	89	Medermycin	Benzoisochromanequinone	Antibacterial, Antitumor^129^
PB-70	KY235153	AlnL ketoacyl synthase of *Streptomyces* sp. CM020 (ACI88861)	74	Alnumycin	Naphthoquinone, Benzoisochromanequinone related	Antitumor, Topoisomerase inhibitory^130^
PB-75	KY235154	β-ketoacyl synthase of *S. nogalater* ATCC 27451 (CAA12017)	79	Nogalamycin	Anthracycline	Antibacterial, Antitumor^131^
PB-81	KY235155	β-ketoacyl-ACP synthase homolog of *S. cyanogenus* S136 (AAD13536)	83	Landomycin	Angucycline	Antitumor^132^
Kz-12	KY235157	ChaA β-ketoacyl synthase of *S. chartreusis* HKI-249 (CAH10161)	68	Chartreusin	Aromatic polyketide glycoside	Antibacterial, Antitumor^60^
Kz-13	KY235158	β-ketoacyl synthase of *S. arenae* DSM 40737 (AAD20267)	75	Naphthocyclinone	Naphthoquinone, Isochromanequinone	Antibacterial^133^
Kz-28	KY235159	β-ketoacyl synthase I of *Streptomyces* sp. R1128 (AAG30189)	70	R1128	Anthraquinone	Estrogen receptor antagonist^134^
Kz-55	KY235160	Jadomycin polyketide ketosynthase of *S. venezuelae* ATCC 10712 (AAB36562)	84	Jadomycin B	Angucycline	Antibacterial^135^
Kz-66	KY235161	3-ketoacyl-ACP synthase of *S. griseus* ATCC 49344 (AAQ08916)	70	Fredericamycin	—	Antibacterial, Antifungal, Antitumor^136^
Kz-74	KY235162	BenA β-ketoacyl synthase of *Streptomyces* sp. A2991200 (CAM58798)	71	Benastatin	Pentangular polyketide	Antibacterial, Apoptosis inducer, glutathione-S-transferase inhibitor^137^
**NRPS**						
PB-52	KU721842	NRPS for virginiamycin S of *S. virginiae* MAFF 10-06014 (BAF50720)	40	Virginiamycin	Streptogramin	Antibacterial^138^
PB-64	KY235156	NRPS peptide synthetase of *S. albus* JA3453 (ABS90470)	53	Oxazolomycin	Polyene-type alkaloid	Antibacterial, Antitumor, Antivirus, Ionophore^139^

These genes were translated to amino acid sequences and the secondary metabolite pathway products were identified using DoBISCUIT database. The genes of all the isolates showed similarities to the phylum actinobacteria at the amino acid level. PKS-I sequences shared 56–68% similarity with their closest matches at the amino acid level in BLASTP search. The amino acid sequences of PB-10, PB-32, PB-47, PB-52, PB-64 and Kz-24 matched with the KS domain and are closely related to the gene product involved in concanamycin A, tautomycin, chlorothricin, nanchangmycin, oligomycin and rifamycin production respectively. PKS-II sequences of 20 actinobacteria shared  68–95% similarity with their closest matches at the amino acid level. Their closest match pathway products were predicted as actinorhodin, naphthocyclinone, jadomycin B, landomycin. These compounds belonged to diverse polyketide groups such as glycosides, anthracycline, naphthoquinone, angucycline, benzoisochromanequinone. Highest sequence identity (95%) at amino acid level was observed in PB-66 which was most closely involved in the synthesis of urdamycin. Adenylation domain of NRPS genes was screened to be positive in PB-52 and PB-64. At the amino acid level, NRPS fragments derived from PB-52 and PB-64 showed 40–53% similarity to their closest relatives. The closest match pathway products of PB-52 and PB-64 isolates were analyzed as virginiamycin and oxazolomycin belonging to streptogramin and polyene-type alkaloid group of compounds respectively (Table 3). Interestingly, PB-64 harbors all the three antibiotic biosynthetic genes, i.e. PKS-I, PKS-II and NRPS. This isolate had the ability to inhibit all the test microorganisms during *in vitro* antimicrobial screening.

### Amplified Ribosomal DNA Restriction Analysis (ARDRA)

**Figure 1:**
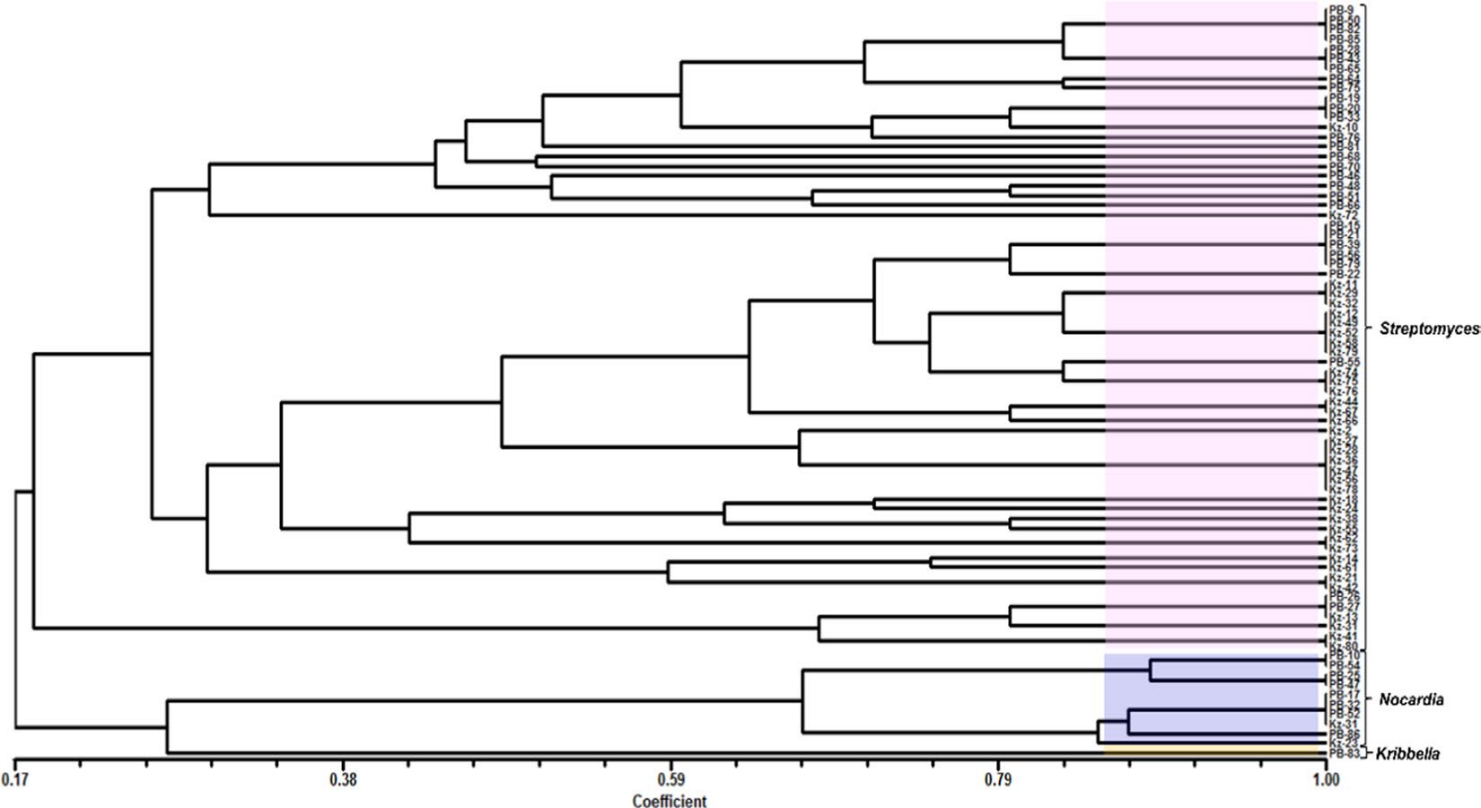
UPGMA dendrogram showing the clustering of 77 antagonistic actinobacteria isolates based on the 16S rDNA generated from ARDRA by Jaccard’s coefficient using NTSYS-pc 2.02e. The scale on the x-axis refers to the similarity coefficient.

The restriction digestion profile of the 77 antagonistic actinobacteria were analysed by ARDRA fingerprinting analysis using HinfI restriction endonuclease. Digestion with Hinf1 showed different restriction banding patterns and the dendrogram was constructed. Fragments which were smaller than 100 bp in size could not be reproducibly visualized and so not considered. Critical analysis of the dendrogram obtained from Unweighted Pair Group Method with Arithmetic Mean (UPGMA) revealed three major clusters consisting of *Streptomyces* sp. *Nocardia* sp. and *Kribbella* sp. Overall analysis of the soil samples (Sample 1–4 and A–E) from the two forest ecosystems, indicated *Streptomyces* as the dominant genus in these habitats (Fig. 1).

### 16S rDNA sequence analysis

**Figure 2:**
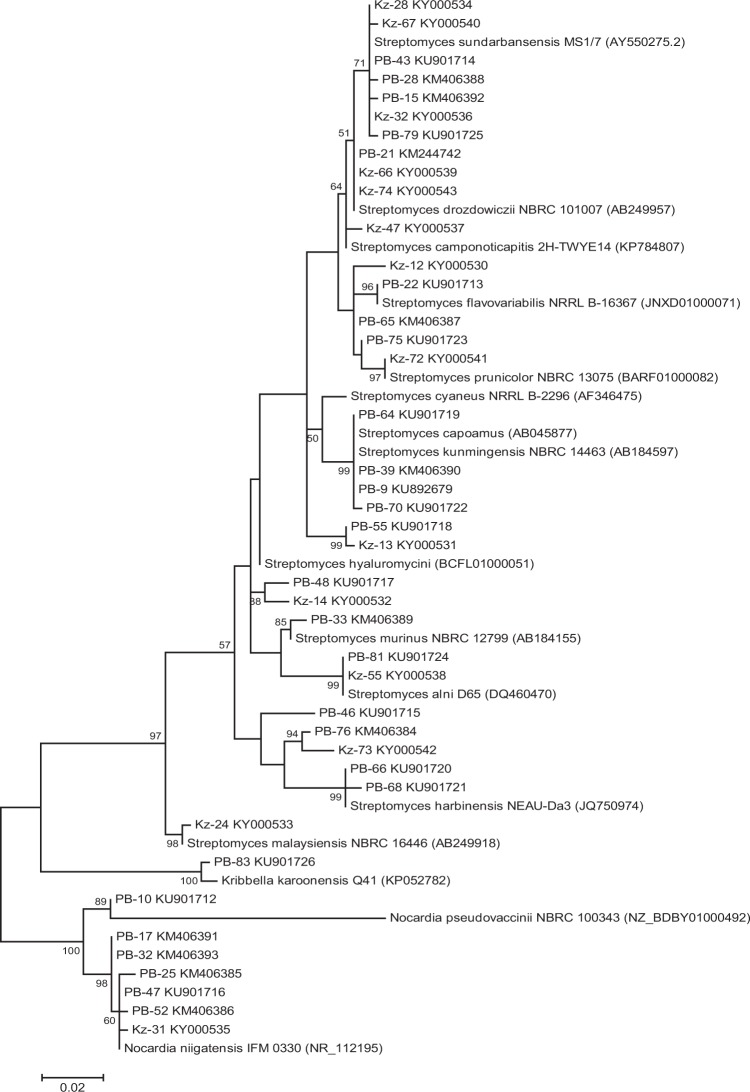
Phylogenetic tree of actinobacteria isolated from forest ecosystems and the closest type strains based on the 16S rDNA sequences by maximum likelihood method using Kimura-2 parameter model. Numbers at branches indicate bootstrap values in 1,000 replicates. Bar, 0.02 substitutions per nucleotide position.

The 16S rDNA of 41 representative actinobacteria (600–1388 bp) exhibited low to high level of sequence similarity (89–100%) to the 16S rDNA of their closest homolog match using EzTaxon database. 4 strains, namely, *Streptomyces* sp. PB-79 (GenBank accession no. KU901725; 1313 bp), *Streptomyces* sp. Kz-28 (GenBank accession no. KY000534; 1378 bp), *Streptomyces* sp. Kz-32 (GenBank accession no. KY000536; 1377 bp) and *Streptomyces* sp. Kz-67 (GenBank accession no. KY000540; 1383 bp) showed very low levels of sequence similarities (~89.5%) to their nearest type strain. Taxonomic identification of the representative isolates revealed that *Streptomyces* was the predominant genus (n = 33), followed by *Nocardia* (n = 7) and *Kribbella* (n = 1). The phylogenetic tree was constructed using MEGA (Molecular Evolutionary Genetics Analysis) version 6 based on maximum likelihood method represented in Fig. 2. The partial 16S rDNA sequences of the actinobacteria have been deposited in GenBank database under the following accession numbers: KM406384-KM406393, KU901712-KU901726, KY000530-KY000543, KM244742 and KU892679.

Kz-24 was selected based on its promising antimicrobial potential against test microorganisms and the partial 16S rDNA sequence (1367 bp) was deposited to NCBI GenBank with accession number KY000533. The strain indicated maximum 16S rDNA similarity (96.2%) with *S. malaysiensis* NBRC 16446 (AB249918). The phylogenetic tree also showed the closest similarity to *S. malaysiensis* based on maximum likelihood method (Fig. 2). The phenotypic and genomic data showed that Kz-24 belonged to genus *Streptomyces* and was thus, referred to as *Streptomyces* sp. strain Kz-24.

### Minimum inhibitory concentration (MIC) of EA-Kz-24

Broth dilution method was used to determine MIC of EA-Kz-24 ranging from 100-0.024 μg/mL against all test microorganisms (Table 4). EA-Kz-24 exhibited lowest MIC against MRSA ATCC 43300 and *C. albicans* MTCC 227 (0.024 μg/mL) whereas highest was recorded against *S. marcescens* MTCC 97 (50 μg/mL). According to Clinical and Laboratory Standards Institute (CLSI) recommendations for MIC, *S. marcescens* MTCC 97 were found to be resistant to EA-Kz-24 (MIC: 50 μg/mL), as ≤8 μg/mL was taken as susceptible, ≤16 μg/mL as intermediate and ≥32 μg/mL as resistant.

**Table 4:** MIC (µg/mL) of EA-Kz-24 by broth dilution method.

Test microorganisms	MIC of EA-Kz-24 (µg/mL)	MIC of Rif (µg/mL)	MIC of Strep (µg/mL)	MIC of Amp B (µg/mL)
Gram-positive bacteria				
***S. aureus*** **MTCC 96**	>0.098	>1.95	>6.25	NA
***S. aureus*** **MTCC 3160**	>0.195	>1.95	>12.5	NA
***S. epidermidis*** **MTCC 435**	>0.78	>3.125	>6.25	NA
***B. subtilis*** **MTCC 441**	>0.048	>1.95	>6.25	NA
***B. cereus*** **MTCC 1272**	>0.78	>6.25	>12.5	NA
***B. megaterium*** **MTCC 8075**	>0.048	>3.12	>3.12	NA
***M. luteus*** **MTCC 1538**	>0.39	>0.97	>6.25	NA
**MRSA ATCC 43300**	>0.024	>25	>50	NA
Gram-negative bacteria				
***E. coli*** **MTCC 40**	>0.39	>6.25	>3.12	NA
***E. coli*** **MTCC 739**	>1.56	>50	—	NA
***S. marcescens*** **MTCC 97**	>50	>12.5	>3.12	NA
***K. pneumoniae*** **MTCC 3384**	>6.25	>25	—	NA
***K. pneumonia*** **ATCC 13883**	>25	>50	—	NA
***P. aeruginosa*** **MTCC 741**	>3.125	>25	>25	NA
***P. aeruginosa*** **MTCC 424**	>12.5	—	>12.5	NA
***P. aeruginosa*** **MTCC 2582**	>3.125	>50	>25	NA
***P. vulgaris*** **MTCC 426**	>25	>25	>6.25	NA
Yeast				
***C. albicans*** **MTCC 227**	>0.024	NA	NA	>0.97
***C. tropicalis*** **MTCC 2208**	>0.78	NA	NA	>0.48
***C. albicans*** **ATCC 10231**	>0.098	NA	NA	>1.95

Rif: Rifampicin (antibacterial agent); Strep: Streptomycin (antibacterial agent); Amp B: Amphotericin B (antifungal agent); NA: not applicable; -: No activity.

### SEM analysis

**Figure 3:**
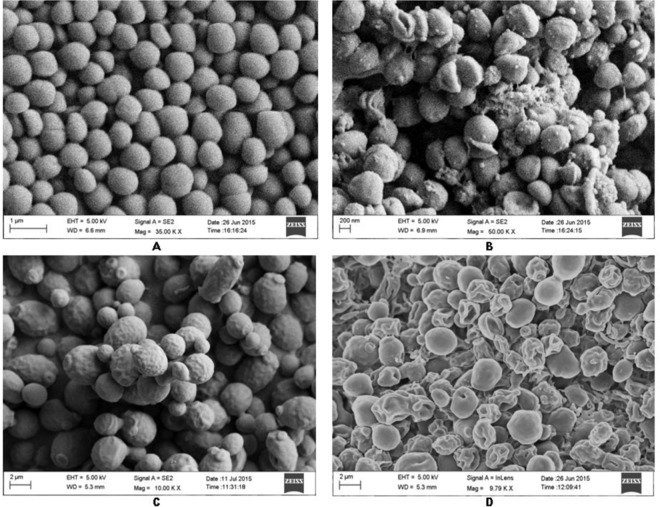
Scanning electron micrograph showing the effect of 1×MIC EA-Kz-24 against MRSA ATCC 43300 (**A**) without treatment, (**B**) treatment with EA-Kz-24; and against *C. albicans* MTCC227 (**C**) without treatment, (**D**) treatment with EA-Kz-24.

SEM was performed for the assessment of antibacterial and anti-candidal activity of EA-Kz-24 against MRSA ATCC 43300 and *C. albicans* MTCC 227. SEM indicated significant morphological changes in the cells of the test microorganisms including cell deformity and shrinkage leading to prominent loss and integrity of cell shape after treatment with 1 × MIC EA-Kz-24. 10% DMSO-treated control cells appeared smooth with intact cell surface (Fig. 3).

### GC-MS analysis

Chemical composition of EA-Kz-24 was characterized with GC-MS. Based on the retention time, molecular weight and molecular formula, thirteen chemical compounds were identified by comparing their mass spectra with the NIST library as shown in Table 5. The peak area of the compound is directly proportional to its antimicrobial metabolite quantity (See Supplementary Fig. S4).

**Table 5:** Chemical compounds detected in EA-Kz-24 by GC-MS analysis.

Sl no	Compound name	RT (min)	MW (g/mol)	Formula	Abundance%	Nature of compound	Activity
1	(Z)-3-Tridecene	18.27	182.34	C_13_H_26_	1.23	Alkene	No activity reported
2	3,5-bis(1,1-dimethylethyl)-phenol	19.93	206.32	C_14_H_22_O	6.72	Phenolic compound	Antimicrobial^24^
3	Benzoic acid,4-ethoxy-ethyl ester	20.13	194.22	C_11_H_14_O_3_	1.66	Benzoic acid ester	Antimicrobial^91^
4	(Z)-3-Tetradecene	20.94	196.37	C_14_H_28_	2.61	Alkene	Antimicrobial^88^
5	Dodecyl acrylate	22.13	240.38	C_15_H_28_O_2_	12.39	Ester	Antibacterial^90^
6	Propanoic acid, decyl ester	22.21	214.34	C_13_H_26_O_2_	3.06	Ester	No activity reported
7	N-acetyl-3-methyl-1,4-diazabicyclo[4.3.0]nonan-2,5-dione	22.56	210.22	C_10_H_14_N_2_ O_3_	2.01	Piperazine	No activity reported
8	Hexahydro-pyrrolo[1,2-a]pyrazine-1,4-dione	23.12, 29.46	154.16	C_7_H_10_N_2_O_2_	22.91	Pyrrolizidine	Antimicrobial^140^
9	(E)-9-Octadecene	23.32	252.47	C_18_H_36_	1.71	Alkene	Antimicrobial^87,89^
10	Hexahydro-3-(2-methylpropyl)-pyrrolo[1,2-a]pyrazine-1,4-dione	23.77, 24.07, 24.83, 25.01, 25.11, 25.18	210.27	C_11_H_18_N_2_O_2_	37.27	Pyrrolizidine	Antimicrobial^140^
11	Propanoic acid,3-mercapto-dodecyl ester	25.74	274.46	C_15_H_30_O_2_S	1.01	Ester	No activity reported
12	3-(phenylmethyl)-2,5-piperazinedione	27.58	204.22	C_11_H_12_N_2_O_2_	1.41	Piperazinedione	No activity reported
13	Hexahydro-3-(phenylmethyl)-pyrrolo[1,2-a]pyrazine-1,4-dione	29.07	244.28	C_14_H_16_N_2_O_2_	6.00	Pyrrolizidine	Antimicrobial^85,140^

RT is retention time; MW is molecular weight of compounds.

## Discussion

Actinobacteria have been recognized as the most proliferant producers of natural bioactive compounds like antimicrobials, wide range of enzymes and valuable secondary metabolites with incredible diversity of biological activities^27–29^. Our primary goal was to study the functionality of culturable actinobacteria from the Indo-Burma mega-biodiversity hotspot with key emphasis towards understanding their biosynthetic potential.

In total, 107 presumptive actinobacteria were isolated from nine varied soil samples collected from Pobitora Wildlife Sanctuary and Kaziranga National Park of Assam, India. Forest is considered as a wealthy biological diversity with millions of animals, plants and microorganisms^30,31^. Soil samples are known to be rich in organic matter and microorganisms where most of the biological activities occur. A total of 34 actinobacteria were isolated from leaf litter soil and tree rhizosphere soil each, 26 from sediment soil and only 13 isolates from grass rhizosphere soil. The pH range of the soil samples was found to be in the range 4.5–6.0 implying to acidic soil pH of the forest ecosystems. Forest soils are previously reported to be of low pH^32^. Actinomycetes isolation agar, Streptomyces agar and GLM agar were used for actinobacteria isolation which was reported previously^12,19^. Maximum numbers of actinobacteria (n = 52; 49%) were recovered from Actinomycetes isolation agar as this media contains glycerol and asparagine which most actinobacteria use as a source of carbon and nitrogen respectively. Additionally, it also contains sodium propionate which makes it most suitable for the isolation of actinobacteria because it acts as an antifungal agent^19^. The use of amending the isolation media with amphotericin B and rifampicin has been confirmed to be a good strategy for promotion of growth of slow growing actinobacteria in the absence of fast growing contaminants^19,33,34^.

Morphological differentiation of all the 107 actinobacteria was done based on their colony morphology, aerial and substrate mycelium colour and diffusible pigment production. The chromogenicity of aerial mycelium is a significant character for grouping of actinobacteria^35^. Furthermore, 38 actinobacteria produced diffusible pigment which is considered as a characteristic feature for identification and classification of *Streptomyce*s^36^.

Actinobacteria have been screened from diverse habitats for the search of novel bioactive compounds from last few years^37–39^. Yet there are very few reports regarding the exploration of actinobacteria from forest ecosystems for production of bioactive compounds. In the preliminary screening by spot inoculation method, 77 actinobacteria (72%) were found to be potential antagonists against at least one of the test microorganisms. A total of 51 isolates exhibited antagonistic action against *S. aureus* MTCC 96, 49 isolates against MRSA ATCC 43300, 59 isolates against *E. coli* MTCC 40 and 60 isolates against *C. albicans* MTCC 227. As reported by Berdy^4^, while many antibiotic compounds display antimicrobial activity against Gram-positive bacteria, only about 1.5% of those are effective against Gram-negative bacteria. There are limited effective antifungal agents for treating life-threatening fungal infections, which is a major challenge to the pharmaceutical industry^40^. In the present study, the result of significant antimicrobial activity against Gram-negative bacteria and fungus strongly suggests that forest ecosystems of Assam are a good source of antagonistic actinobacteria exhibiting promising antimicrobial activity.

Out of 77 antagonistic actinobacteria, 63, 56, 53, 59 and 58 number of isolates produced amylase, cellulase, protease, lipase and esterase enzymes respectively. Interestingly, 24 isolates produced all the five enzymes. Ramesh and Mathivanan^41^ and Meena *et al*.^42^ isolated actinobacteria from marine sources with multi-enzyme activity. As per the findings of Ramesh and Mathivanan^41^, they isolated actinobacteria from marine sources and found that majority of the isolates produced lipase which plays an important role in the degradation of polymers in oceans for adaptation in the extreme environment. However, in this study, 56 actinobacteria isolated from the forest ecosystems produced cellulase. The population of cellulase producing actinobacteria is reasonably high in forest floors and soil for the purpose of soil cycling and decomposition of tough plant materials and woody stems. These actinobacteria are largely responsible for the breakdown of large biopolymers like cellulose, hemicellulose, lignin and chitin^32,43,44^.

Biosynthetic gene clusters PKS-I, PKS-II and NRPS play a fundamental role in the biosynthesis of microbial natural products^45^. Out of the 77 actinobacteria exhibiting antimicrobial activity, 24 isolates tested positive for at least one of these biosynthetic genes. This result indicated that 31% of the isolates with antimicrobial potential from the forest ecosystems possessed one of the biosynthetic genes. A total of 6 isolates (8%) were found positive for the presence of PKS-I genes while 20 isolates (26%) were positive for PKS-II genes and NRPS genes were detected in only 2 isolates (3%). These findings were in concurrence with the reports of Lee *et al*.^46^, who isolated actinobacteria from mangrove forest soil in Malaysia and reported that PKS-II genes were found to be most frequent among the actinobacteria endowed with antimicrobial activity in forest ecosystems, followed by PKS-I and NRPS genes. Lack of amplification of biosynthetic gene sequences in some of the isolates may be due to absence of these genes in their genome^47,48^. The absence of PKS and NRPS genes does not detriment the antagonistic activity of the isolates, signifying that additional biosynthetic mechanisms or types of bioactive agents may be involved in the production of antimicrobial activity^49^. Genus *Streptomyces* and *Nocardia* are previously reported as recognized producers of polyketides and nonribosomal peptides^24,38^. Earlier reports confirm that *Streptomyces* strains possess multiple copies of PKS and NRPS genes the functions of which are still not explored fully^50–52^. In the antimicrobial screening, *Streptomyces* sp. PB-64 exhibited bioactivity against all the test microorganisms and interestingly it indicated the presence of all the three biosynthetic genes i.e. PKS-I, PKS-II and NRPS. Challis^53^ reported that microbial genome sequences harvest many orphan or cryptic biosynthetic gene clusters which have the capability to direct the synthesis of novel, structurally complex natural products. The secondary metabolite pathway products of these biosynthetic gene clusters were predicted using DoBISCUIT database^54^. In recent years, prediction of chemical classes have been applied effectively for the discovery of type I and type II polyketides and nonribosomal peptides^38,55,56^. The top BLAST match of the PKS-I gene sequences of the six actinobacteria isolates PB-10, PB-32, PB-47, PB-52, PB-64 and Kz-24 yielded different pathway products such as concanamycin A, tautomycin, chlorothricin, nanchangmycin, oligomycin and rifamycin respectively classified under macrocyclic lactone, tetronic acid derivative, ansamycin and polyether group of compounds. These compounds are reported to be antimicrobials in nature effective against a wide range of bacterial and fungal pathogens along with their antitumor properties^27,57,58^. Top BLAST match of the PKS-II gene sequences of the actinobacteria isolates PB-9, PB-10, PB-15, PB-22, PB-33, PB-47, PB-48, PB-64, PB-65, PB-66, PB-68, PB-70, PB-75, PB-81, Kz-12, Kz-13, Kz-28, Kz-55, Kz-66 and Kz-74 yielded significant aromatic polyketides such as naphthocyclinone, jadomycin B, urdamycin, nogalamycin, actinorhodin. classified under the angucycline, anthracycline, naphthoquinone group of compounds. The compounds are reported to possess excellent antimicrobial activity against human disease causing pathogens and antitumor property^8,59,60^. Top BLAST match pathway products of the NRPS gene sequences of PB-52 and PB-64 yielded virginiamycin and oxazolomycin classified under streptogramin and polyene-type alkaloid group of compounds. Virginiamycin is a PKS/NRPS hybrid showing closest amino acid sequence similarity to PB-52 isolate which indicated the presence of both PKS-I and NRPS during gene-targeted PCR amplification. Additionally, oxazolomycin is also a PKS/NRPS hybrid showing closest amino acid sequence similarity to PB-64 isolate and interestingly, this isolate was positive for the presence of all the three biosynthetic genes. These compounds are reported to be antibacterial in nature^61,62^.

16S rDNA-ARDRA is an excellent molecular genome typing method for classification of actinobacteria at the genus level^63^. Comparative diversity analysis of the 77 antagonistic actinobacteria through ARDRA using Hinf1 revealed significant difference among the isolates indicating its true diversity study. The genetic variation among the isolates might be due to mutation or recombination^64,65^. Natural populations of soil bacterium may exhibit genetic diversity due to variable habitat conditions and soil properties^66^. Also, the genetic composition and diversity of actinobacteria is influenced by variety in frequency and intensity of competition among locations of isolation^67^. ARDRA has proved its use in differentiating bacterial species within same genus and bacterial strains within same species^68,69^. Genetic diversity of the forests soil consisted of *Streptomyces*, *Nocardia* and *Kribbella* sp. which was determined by sequencing of 16S rDNA. Based on ARDRA fingerprinting analysis, strong antimicrobial activity and presence of antibiotic biosynthetic genes (PKS-I, PKS-II and NRPS), 41 isolates were selected as representatives and partially identified by 16S rDNA sequencing. BLAST search results implied that these 41 isolates belonged to the genus *Streptomyces* (33 isolates), *Nocardia* (7 isolates) and *Kribbella* (1 isolate). From the sequencing result, it was clear that *Streptomyces* was found to be the dominant genus in the soil of protected forest ecosystems which was also reported previously^12,19,70,71^.

Kz-24 was selected based on its promising antimicrobial potential against the test microorganisms. MIC of EA-Kz-24 support the popular notion that antimicrobial metabolites extracted from *Streptomyces* sp. at very low concentrations can be one of the finest sources of potent antimicrobials for treatment of infectious diseases especially those caused by clinically resistant pathogens, such as *P. aeruginosa*, MRSA, *C. albicans*^72^. Our finding is similar with the result of Kumar *et al*.^73^ where the crude ethyl acetate extract of *S. lavendulae* SCA5 showed potent antimicrobial action with an MIC of 125 μg/mL against bacteria and MIC against fungi was 31.25 μg/mL. Kz-24 showed 96.2% 16S rDNA sequence similarity with *S. malaysiensis* NBRC 16446 (AB249918). The aerial mycelium of Kz-24 was brown in colour in GLM agar containing yeast extract/malt extract while *S. malaysiensis* NBRC 16446 was repoted to be dark grey in a medium with same composition^74^. *S. malaysiensis* was previously reported to exhibit strong antifungal activity and low antibacterial activity against *B. subtilis* NCIB 3610^74–76^. This is the first report on its antibacterial activity against *E. coli* MTCC 40.

SEM experiments further confirmed that the strong antimicrobial activity of EA-Kz-24 led to significant morphological changes in the selected test microorganisms leading to cell shrinkage and cytosolic loss. These results are in symmetry with the findings of Sharma *et al*.^24^, Supaphon *et al*.^77^, Nurkanto and Julistiono^78^.

Actinobacteria are significant producers of bioactive secondary metabolites with different biological activity. There are many reports available for the use of GC-MS to analyze microbial metabolites chemically^79–81^. In this study, GC-MS analysis was performed on EA-Kz-24 and thirteen chemical compounds with different retention time and abundance were detected. The compounds identified were esters, alkenes, a phenolic compound, diketopiperazine and pyrrolidinopiperazine. Phenolic compounds are known to be powerful antimicrobials and antioxidants because they can reduce free radicals by hydrogen-donating ability^82^. Studies led by Balachandran *et al*.^83^ and Kumar *et al*.^84^ exhibited maximum antimicrobial action with highest phenolic compounds in GC-MS fractions. The antimicrobial activity of 3,5-bis(1,1-dimethylethyl)-phenol by *Nocardia* sp. PB-52 was reported for the first time by our group^24^. The pyrrolizidine compounds present in EA-Kz-24 included hexahydro-pyrrolo[1,2-a]pyrazine-1,4-dione (22.91%), hexahydro-3-(2-methylpropyl)-pyrrolo[1,2-a]pyrazine-1,4-dione (37.27%) and hexahydro-3-(phenylmethyl)- pyrrolo[1,2-a]pyrazine-1,4-dione (6%). Previous findings conclude that these compounds possess promising antimicrobial activity^85,86^. These compounds could be the key contributors for potent antimicrobial action of EA-Kz-24. A subset of researchers reported the antagonistic potential of alkenes such as (Z)-3-tetradecene and (E)-9-octadecene against an array of test pathogens^87–89^ but (Z)-3-tridecene has not been reported for any antimicrobial activity till date. Manilal *et al*.^90^ reported antibacterial action of dodecyl acrylate against multidrug-resistant clinical pathogens by *Falkenbergia hillebrandii*, a red algae. Hussain and Kumaresan^91^ reported the antifungal nature of benzoic acid,4-ethoxy-ethyl ester. The compounds detected in GC-MS are well known for their ability to inhibit the growth and proliferation of test microorganisms and together may be responsible for the broad-spectrum antimicrobial activity of EA-Kz-24. However, till date, there have been no report on the antimicrobial effect of propanoic acid decyl ester, propanoic acid,3-mercapto-dodecyl ester, 3-(phenylmethyl)-2,5-piperazinedione and N-acetyl-3-methyl-1,4-diazabicyclo[4.3.0]nonan-2,5-dione which are the key components of EA-Kz-24. Propanoic acid decyl ester and propanoic acid,3-mercapto-dodecyl ester are esters in nature, 3-(phenylmethyl)-2,5-piperazinedione is a piperazinedione compound and N-acetyl-3-methyl-1,4-diazabicyclo[4.3.0]nonan-2,5-dione lies in the piperazine group of compounds. These compounds together constitute 7.49% of the total constituents present in EA-Kz-24. Previous report by Manilal *et al*.^90^ reported strong antimicrobial activity of ester compounds. Niku-Paavola *et al*.^92^ reported that a piperazinedione compound, 3-(2-methylpropyl)-2,5-piperazinedione, from *Lactobacillus plantarum* strongly inhibited the growth of *Pantoea agglomerans* and *Fusarium avenaceum*. Musthafa *et al*.^93^ reported the effect of 2,5-piperazinedione in reducing the production of quorum sensing dependent factors in *Pseudomonas aeruginosa* PAO1 both *in vivo* and *in vitro*. According to the reports by Jain *et al*.^94^, 1-(4-chlorophenyl)-1-propyl piperazine and 1-(4-methylphenyl)-1-propyl piperazine exhibited excellent inhibitory activity against *S. aureus* and *P. aeruginosa* respectively. Jadhav *et al*.^95^ reported strong antibacterial and antifungal activity by different piperazine group of compounds. Thus, it can be concluded that propanoic acid decyl ester, propanoic acid,3-mercapto-dodecyl ester, 3-(phenylmethyl)-2,5-piperazinedione and N-acetyl-3-methyl-1,4-diazabicyclo[4.3.0]nonan-2,5-dione present in EA-Kz-24 might have a significant role to play in its inhibitory effect against a wide range of test microorganisms which is reported for the first time. The combinatorial effect of bioactive compounds found in GC-MS analysis was demonstrated previously^79–81^. We, therefore, suggest that these compounds might be the key contributing factor for the antimicrobial action of EA-Kz-24. The forest ecosystems of biodiversity hotspots represent diverse and largely underexplored ecosystem for the isolation of actinobacteria producing effective antimicrobial compounds. It can be inferred from our findings that actinobacteria can be the fundamental sources for the discovery of valuable antibiotic compounds of high industrial and commercial values for human welfare.

## Materials and Methods

### Sample collection and measurement of soil pH

**Figure 4:**
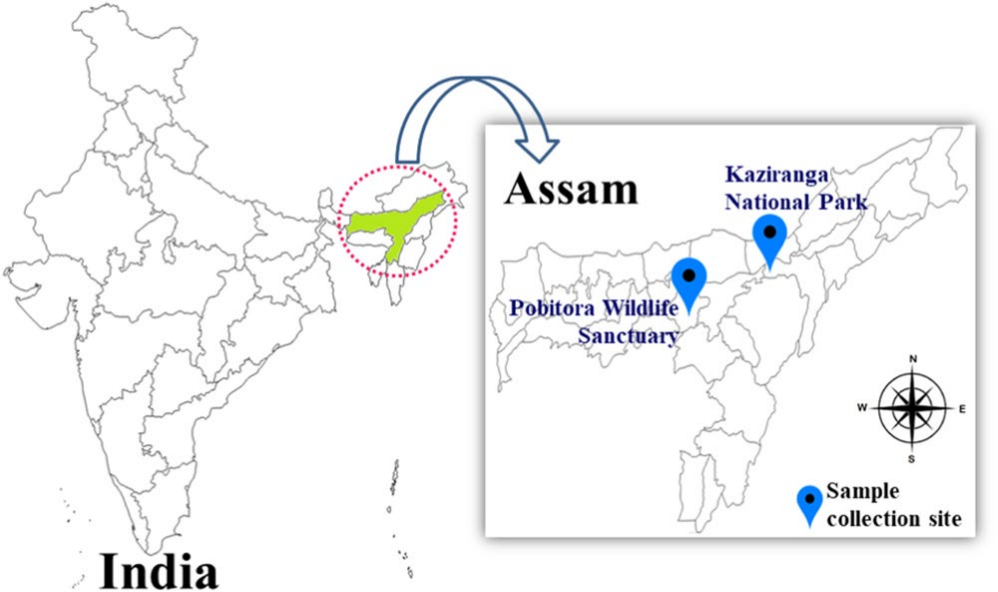
Sampling sites for isolation of actinobacteria are denoted. Soil samples were collected from different environmental sites of Pobitora Wildlife Sanctuary and Kaziranga National Park of Assam, India. (Map has been adapted from https://d-maps.com/index.php?lang = en).

Soil samples were collected from Pobitora Wildlife Sanctuary and Kaziranga National Park of Assam, India (Fig. 4). Soil samples each weighing ∼50 g were collected randomly in sterile zip-lock plastic bags within 50 m^2^ area from a depth of 5 to 20 cm after removing the top soil. Four samples from each site were bulked and homogenized to prepare composite samples. pH of the soil samples was also measured^41^.

### Selective isolation and preliminary identification of actinobacteria

The composite soil samples were suspended in physiological saline (NaCl 9 g/L), thoroughly homogenized by stirring and serial dilutions up to 10^−4^ were plated on three isolation media: Actinomycetes isolation agar, Streptomyces agar and GLM agar (Yeast extract, 3 g; malt extract, 3 g; peptone Type I, 5 g; starch, 10 g; agar, 20 g; distilled water, 1000 mL) supplemented with amphotericin B (75 µg/mL) and rifampicin (2.5 µg/mL)^19,24^. The inoculated plates were incubated at 28 °C and examined regularly for the appearance of actinobacteria colonies until 4 weeks.

The pure isolates were grouped according to their colony morphology, colour of aerial and substrate mycelium, colour of diffusible pigments and spore chain morphology^96,97^. The spore chain morphological features were observed by light microscopy.

### Screening for antimicrobial potential

The following test microorganisms were used for the experiment. *Staphylococcus aureus* MTCC 96, *S. aureus* MTCC 3160, *S. epidermidis* MTCC 435, MRSA ATCC 43300, *Micrococcus luteus* MTCC 1538, *Bacillus cereus* MTCC 1272, *B. subtilis* MTCC 441, *B. megaterium* MTCC 8075 (Gram-positive bacteria); *Escherichia coli* MTCC 40, *E. coli* MTCC 739, *Klebsiella pneumoniae* MTCC 3384, *K. pneumoniae* ATCC 13883, *Serretia marcescens* MTCC 97, *Proteus vulgaris* MTCC 426, *Pseudomonas aeruginosa* MTCC 741, *P. aeruginosa* MTCC 2582, *P. aeruginosa* MTCC 424 (Gram-negative bacteria); *Candida albicans* MTCC 227, *C. albicans* ATCC 10231 and *C. tropicalis* MTCC 2208 (yeasts). MTCC cultures were procured from Microbial Type Culture Collection (CSIR-IMTECH), India and ATCC cultures from HiMedia, India.

The preliminary antimicrobial screening was done by spot inoculation method^98^ using four test microorganisms. Gram-positive bacterium: *Staphylococcus aureus* MTCC 96; Gram-positive resistant bacterium: Methicilin resistant *Staphylococcus aureus* (MRSA) ATCC 43300; Gram-negative bacterium: *Escherichia coli* MTCC 40; yeast: *Candida albicans* MTCC 227. The diameter of inhibition zones was determined after 24 hrs^99^. The experiment was repeated for three times.

The actinobacteria found to be promising in the preliminary antimicrobial screening were subjected to secondary screening by disc diffusion method (Bauer *et al*., 1966). The following test microorganisms were used for this study: *S. aureus* MTCC 96, *S. epidermidis* MTCC 435, *Bacillus subtilis* MTCC 441, *Micrococcus luteus* MTCC 1538, MRSA ATCC 43300 (Gram-positive bacteria); *E. coli* MTCC 40, *Serretia marcescens* MTCC 97, *Klebsiella pneumoniae* MTCC 3384, *Pseudomonas aeruginosa* MTCC 741 (Gram-negative bacteria); and *C. albicans* MTCC 227 (yeast). Crude antimicrobial extracts were recovered from the culture filtrate by solvent extraction using ethyl acetate (1:1, v/v). The diameters of zone of inhibition were determined after 24 hours. All samples were assayed in triplicate.

### Screening for the production of extracellular enzymes

All the 77 antagonistic actinobacteria were screened qualitatively for the production of 5 important extracellular enzymes, i.e. amylase, cellulase, protease, lipase and esterase. Each actinobacteria was spot inoculated on agar plates amended with the respective substrates such as starch, carboxyl methyl cellulose, casein and Tween 80 and Tween 20 and was incubated for up to 10 days at 28 °C^100–103^.

### PCR amplification of biosynthetic genes PKS-I, PKS-II and NRPS and analysis of biosynthetic genes for prediction of chemical classes

Genomic DNA isolation of actinobacteria and PCR amplification of PKS-I and NRPS was performed as described previously^24,104^. Degenerate primers KSαF (5′-TSG CST GCT TCG AYG CSA TCA-3′) and KSαR (5′-TGG AAN CCG CCG AAB CCG CT-3′) targeting ketosynthase gene of the minimal PKS cluster were used for amplification of PKS-II^105^. PCR reactions were performed in a final volume of 50 μl in Proflex PCR System (Applied Biosystems, USA). The reaction mixture comprised of template DNA (50 ng), each dNTP (0.2 mM), 1X Taq DNA polymerase buffer, MgCl_2_ (1.5 mM), each primer (0.2 µM) and 1 U Taq DNA polymerase. The profile used for amplification of PKS-II genes were programmed as: initial denaturation at 94 °C for 5 mins; followed by 35 cycles at 95 °C for I min, 65 °C for 1 min, 72 °C for 2 mins and a final extension at 72 °C for 10 mins. The size of the amplicons was 613 bp (KSαF/ KSαR). The amplified products were determined by 1.8% (w/v) agarose gel electrophoresis and partially sequenced by automated DNA sequencer at Scigenome Labs Pvt. Ltd. (India).

The nucleotide sequences of PKS-I, PKS-II and NRPS were translated into amino acid sequences using the web tool ORF FINDER (http://www.ncbi.nlm.nih.gov/ projects/gorf/). The deduced amino acid sequences were used as queries to search related gene products in the NCBI and DoBISCUIT (Database of BIoSynthesis clusters CUrated and InTegrated, http://www. bio.nite.go.jp/pks/)^54^ using the BLASTP algorithm^106^ with default parameters. The secondary metabolites pathway products of these biosynthetic genes were identified using DoBISCUIT.

### ARDRA

16S rDNA PCR amplification was carried out as previously described^24,107^. ARDRA was performed for the identification of number of polymorphic groups and then select the representative actinobacteria isolates among these groups^108^. 50 ng of purified 16S rDNA PCR products were digested with 1.5 U of Hinf1^109,110^ following the manufacturer’s protocol (New England Biolabs, UK). The mixture was incubated at 37 °C for 4 hours. Fully digested restriction fragments together with 100 bp and 1 kb markers were resolved by 2% (w/v) agarose gel electrophoresis at 100 volts for 90 min containing 10 μg/mL ethidium bromide. Different ARDRA banding patterns were observed and the isolates were grouped accordingly. ARDRA banding pattern in binary data was graded visually where “1” indicated the presence of band and “0” indicated for absence of band. This binary data was useful for construction of the dendrogram. Similarities among these actinobacteria were calculated by Jaccard’s coefficient in the SimQual program. Similarity index matrix was used to cluster the actinobacteria with SAHN tool based on UPGMA method and the TreePlot program of NTSYS-pc 2.02e software package^111^.

### Identification of actinobacteria by 16S rDNA sequence analysis

Based on the antimicrobial potential, presence of biosynthetic genes and ARDRA, actinobacteria were identified based on the 16S rDNA sequencing using the facility at Scigenom Labs Pvt. Ltd. (India) and Molbiogen (India). Identification of nearest phylogenetic neighbours of sequenced 16S rDNA was carried out using EzTaxon database (http://www. eztaxon. org/)^112^. The 16S rDNA gene sequences used in the phylogenetic analysis was retrieved from NCBI GeneBank. The aligned sequences were used to reconstruct the phylogeny using maximum likelihood method algorithm by MEGA version 6^113^. Bootstrap analysis carried out with 1000 replications determined the support of each clade^114^.

### Determination of MIC of Kz-24

MIC of Kz-24 was performed according to CLSI^115^ and Andrews^116^ using broth dilution method with slight modifications. To 5 mL of Mueller Hinton broth (for bacterial test organisms) and Sabouraud dextrose broth (for yeasts), 1×10^5^cfu/mL inoculum of test microorganisms (log phase culture) was added in different test tubes and incubated at 37 °C for bacteria (24 hours) and 25 °C for yeasts (48 hours). 10% DMSO was used to dissolve EA-Kz-24 (1 mg/mL) and the extract was prepared for MIC screening with two fold serial dilution (100-0.024 μg/mL). MIC was determined in presence of EA-Kz-24 after 24–48 hours. 10 μL test microorganisms were spread on Mueller Hinton agar /sabouraud dextrose agar plates and observed after 24–48 hours for MIC determination. Control with no antimicrobial agent was turbid (negative control) and the control with standard antibiotic such as rifampicin, streptomycin and amphotericin B was clear (positive control).

### Study of antimicrobial effect of EA-Kz-24 by SEM analysis

EA-Kz-24 was studied by SEM for its potential effects on MRSA ATCC 43300 and *C. albicans* MTCC 227 according to Sharma *et al*.^24^. Test microorganisms were treated with 1×MIC EA-Kz-24.

### GC-MS analysis of EA-Kz-24

GC-MS was used for the detection of chemical compounds present in EA-Kz-24 as previously described^24^. The peaks were identified by comparing the mass spectra with the National Institute of Standards and Technology (NIST, USA) library.

### Data analysis

All experiments were conducted in triplicate and repeated three times. The data were presented as the mean of three replicates ± standard deviation of mean. Duncan’s multiple range test was done using SPSS (SPSS 18.0, SPSS Inc., Chicago, IL, USA) in the antimicrobial activity data to compare that the sample means differ at a significant level *P* < 0.05 from each other^117^. Venn diagram is used for the representation of enzymes production by actinobacteria by using multiple dataset analysis features of VENNTURE software^118^.

## Supplementary information

Supplementary Information.

## Data Availability

All data generated or analysed during this study are included in this published article (and its Supplementary Information files).
