# Supplementary material for: Antimicrobial biosynthetic potential and diversity of culturable soil actinobacteria from forest ecosystems of Northeast India
Source: Sci Rep. 2020 Mar 5;10:4104. doi: 10.1038/s41598-020-60968-6 (PMC7057963; doi:10.1038/s41598-020-60968-6)
Supplement: Supplementary file 1 — Supplementary Information. [file 41598_2020_60968_MOESM1_ESM.pdf]

Antimicrobial biosynthetic potential and diversity of culturable soil actinobacteria from forest ecosystems of Northeast India

Priyanka Sharma[1,2] & Debajit Thakur[2]*

[1]Malaria Drug Discovery Laboratory, International Centre for Genetic Engineering and Biotechnology (ICGEB), Aruna Asaf Ali Marg, New Delhi- 110067, India

[2]Microbial Biotechnology Laboratory, Life Sciences Division, Institute of Advanced Study in Science and Technology (IASST), An Autonomous Institute under Department of Science and Technology (Govt. of India), Paschim Boragaon, Garchuk, Guwahati-781035, Assam, India.

*** Corresponding author:**
E-mail address: debajitthakur@yahoo.co.uk

Supplementary Table S1. Isolation source and characterization of actinobacteria isolated from Pobitora Wildlife Sanctuary and Kaziranga National Park of Assam, India based on the colour series

Sl. no.	Isolate code	Isolation site	Sample no.	Colony morphology	Colour of aerial mycelium	Colour of substrate mycelium	Diffusible pigment	Colour series
1.	PB-4	PWS	1 (GRS)	Circular, flat	Light pink	Orange	ND	Variable
2.	PB-9	PWS	1 (GRS)	Curled, flat	Greenish grey	Cream	ND	Grey
3.	PB-10	PWS	1 (GRS)	Undulate, flat	White	Yellow	ND	White
4.	PB-12	PWS	1 (GRS)	Circular, flat	Light pink	Orange	ND	Variable
5.	PB-13	PWS	1 (GRS)	Undulate, raised	Light pink	Orange	ND	Variable
6.	PB-14	PWS	1 (GRS)	Curled, convex	White	Cream	ND	White
7.	PB-15	PWS	1 (GRS)	Curled, convex	Creamy white	Brown	Reddish brown	White
8.	PB-16	PWS	1 (GRS)	Entire, pulvinate	White	Orange	Brown	White
9.	PB-17	PWS	1 (GRS)	Circular, flat	Light pink	Orange	Yellowish brown	Variable
10.	PB-19	PWS	1 (GRS)	Punctiform, raised	Whitish pink	Orange	Creamy brown	Variable
11.	PB-20	PWS	1 (GRS)	Circular, flat	Light pink	Cream	ND	Variable
12.	PB-21	PWS	1 (GRS)	Punctiform, flat	Grey	Reddish brown	Reddish brown	Grey
13.	PB-22	PWS	1 (GRS)	Circular, convex	White	Cream	ND	White
14.	PB-25	PWS	2 (LLS)	Undulate, flat	Light pink	Orange	ND	Variable
15.	PB-26	PWS	2 (LLS)	Erose, raised	White	Cream	ND	White
16.	PB-27	PWS	2 (LLS)	Curled, umbonate	Yellow	Brown	Brown	Yellow
17.	PB-28	PWS	2 (LLS)	Entire, convex	White	Cream	ND	White
18.	PB-31	PWS	2 (LLS)	Undulate, raised	White	Cream	ND	White
19.	PB-32	PWS	2 (LLS)	Circular, flat	Light pink	Orange	ND	Variable
20.	PB-33	PWS	2 (LLS)	Entire, flat	Grey white	Cream	Brown	White
21.	PB-35	PWS	2 (LLS)	Circular, convex	White	Cream	ND	White
22.	PB-36	PWS	3 (TRS)	Punctiform, raised	White	Cream	ND	White
23.	PB-37	PWS	3 (TRS)	Punctiform, flat	White	Cream	ND	White
24.	PB-38	PWS	3 (TRS)	Circular, convex	Orange	Orange	ND	Variable
25.	PB-39	PWS	3 (TRS)	Curled, umbonate	Brown	Creamy brown	Light brown	Variable
26.	PB-43	PWS	3 (TRS)	Punctiform, flat	White	Cream	ND	White
27.	PB-44	PWS	3 (TRS)	Circular, raised	White	Cream	ND	White

Sl. no.	Isolate code	Isolation site	Sample no.	Colony morphology	Colour of aerial mycelium	Colour of substrate mycelium	Diffusible pigment	Colour series
28.	PB-46	PWS	3 (TRS)	Undulate, umbonate	Creamy white	Brown	Light brown	White
29.	PB-47	PWS	3 (TRS)	Circular, flat	Light pink	Cream	ND	Variable
30.	PB-48	PWS	3 (TRS)	Circular, raised	Grey	Brown	Brown	Grey
31.	PB-49	PWS	3 (TRS)	Circular, flat	Orange	Orange	ND	Variable
32.	PB-50	PWS	3 (TRS)	Circular, raised	Light blue	Cream	Light brown	Blue
33.	PB-51	PWS	3 (TRS)	Circular, raised	Grey	Cream	ND	Grey
34.	PB-52	PWS	3 (TRS)	Undulate, flat	Orange	Brown	Light brown	Variable
35.	PB-54	PWS	3 (TRS)	Erose, raised	White	Cream	ND	White
36.	PB-55	PWS	3 (TRS)	Filamentous, raised	Dark brown	Blackish brown	ND	Variable
37.	PB-56	PWS	3 (TRS)	Lobate, raised	Light orange	Brown	ND	Variable
38.	PB-57	PWS	3 (TRS)	Circular, raised	Light pink	Orange	ND	Variable
39.	PB-61	PWS	4 (SS)	Circular, convex	White	Yellow	ND	White
40.	PB-64	PWS	4 (SS)	Erose, flat	White	Brown	Dark brown	White
41.	PB-65	PWS	4 (SS)	Curled, flat	White	Cream	ND	White
42.	PB-66	PWS	4 (SS)	Circular, flat	White	Cream	ND	White
43.	PB-68	PWS	4 (SS)	Circular, flat	White	Cream	Brown	White
44.	PB-70	PWS	4 (SS)	Undulate, raised	Creamy white	Light pink	ND	White
45.	PB-72	PWS	4 (SS)	Entire, convex	White	Yellow	ND	White
46.	PB-75	PWS	4 (SS)	Entire, raised	Brownish white	Brown	ND	White
47.	PB-76	PWS	4 (SS)	Undulate, umbonate	Pink-grey	Maroon	ND	Grey
48.	PB-79	PWS	4 (SS)	Undulate, umbonate	Orange	Orange	ND	Variable
49.	PB-81	PWS	4 (SS)	Filamentous, raised	Pink-grey	Cream	Maroon	Grey
50.	PB-82	PWS	4 (SS)	Lobate, umbonate	Pink	Cream	Light brown	Variable
51.	PB-83	PWS	4 (SS)	Curled, convex	White	Cream	ND	White
52.	PB-84	PWS	4 (SS)	Filamentous, convex	White	Brown	ND	White
53.	PB-85	PWS	4 (SS)	Curled, raised	White	Cream	ND	White
54.	PB-86	PWS	4 (SS)	Curled, pulvinate	White	Cream	ND	White
55.	Kz-2	KNP	A (LLS)	Erose, convex	White	Cream	ND	White
56.	Kz-10	KNP	A (LLS)	Entire, convex	Light yellow	Cream	ND	Yellow

Sl. no.	Isolate code	Isolation site	Sample no.	Colony morphology	Colour of aerial mycelium	Colour of substrate mycelium	Diffusible pigment	Colour series
57.	Kz-11	KNP	A (LLS)	Undulate, raised	White	Cream	ND	White
58.	Kz-12	KNP	A (LLS)	Erose, pulvinate	Grey white	Brown	Brown	White
59.	Kz-13	KNP	A (LLS)	Entire, raised	White	Cream	ND	White
60.	Kz-14	KNP	A (LLS)	Entire, umbonate	Grey	Browny black	Brown	Grey
61.	Kz-18	KNP	A (LLS)	Erose, convex	White	Cream	Light brown	White
62.	Kz-21	KNP	A (LLS)	Undulate, flat	Grey	Brown	Brown	Grey
63.	Kz-23	KNP	A (LLS)	Entire, flat	White	Cream	ND	White
64.	Kz-24	KNP	A (LLS)	Undulate, convex	Brown	Cream	ND	Variable
65.	Kz-25	KNP	B (TRS)	Curled, convex	White	Brown	ND	White
66.	Kz-27	KNP	B (TRS)	Entire, raised	White	Cream	ND	White
67.	Kz-28	KNP	B (TRS)	Undulate, umbonate	Grey	Grey	ND	Grey
68.	Kz-29	KNP	B (TRS)	Curled, umbonate	Grey	Black	ND	Grey
69.	Kz-30	KNP	B (TRS)	Undulate, raised	White	Cream	ND	White
70.	Kz-31	KNP	B (TRS)	Entire, flat	Pink	Cream	ND	Variable
71.	Kz-32	KNP	B (TRS)	Undulate, flat	Brown	Brown	Reddish brown	Variable
72.	Kz-34	KNP	B (TRS)	Filamentous, flat	Light brown	Brown	ND	Variable
73.	Kz-35	KNP	B (TRS)	Entire, convex	Pink	Cream	ND	Variable
74.	Kz-36	KNP	C (SS)	Undulate, convex	White	Cream	ND	White
75.	Kz-38	KNP	C (SS)	Entire, flat	Pink	Cream	Light brown	Variable
76.	Kz-41	KNP	C (SS)	Undulate, convex	Grey	Cream	ND	Grey
77.	Kz-42	KNP	C (SS)	Entire, convex	White	Cream	ND	White
78.	Kz-43	KNP	C (SS)	Undulate, raised	White	Cream	ND	White
79.	Kz-44	KNP	C (SS)	Curled, convex	White	Light brown	ND	White
80.	Kz-45	KNP	C (SS)	Undulate, raised	White	Cream	ND	White
81.	Kz-47	KNP	C (SS)	Undulate, pulvinate	White	Cream	ND	White
82.	Kz-49	KNP	C (SS)	Erose, pulvinate	Brown	Brown	Light brown	Variable
83.	Kz-51	KNP	C (SS)	Curled, umbonate	Yellow	Cream	ND	Yellow
84.	Kz-52	KNP	D (TRS)	Entire, raised	White	Cream	ND	White
85.	Kz- 55	KNP	D (TRS)	Undulate, convex	Pink	Pink	Dark pink	Variable

Sl. no.	Isolate code	Isolation site	Sample no.	Colony morphology	Colour of aerial mycelium	Colour of substrate mycelium	Diffusible pigment	Colour series
86.	Kz-56	KNP	D (TRS)	Undulate, pulvinate	Brown	Brown	Light brown	Variable
87.	Kz-57	KNP	D (TRS)	Erose, convex	Grey	Yellow	Yellow	Grey
88.	Kz-58	KNP	D (TRS)	Curled, umbonate	Grey	Brown	Light brown	Grey
89.	Kz-59	KNP	D (TRS)	Undulate, convex	White	Cream	Light brown	White
90.	Kz-61	KNP	D (TRS)	Curled, umbonate	Bluish grey	Black	Grey brown	Grey
91.	Kz-62	KNP	D (TRS)	Entire, umbonate	Purple	Dark brown	Brown	Variable
92.	Kz-63	KNP	E (LLS)	Erose, convex	Pink	Cream	ND	Variable
93.	Kz-64	KNP	E (LLS)	Lobate, umbonate	Grey	Yellow	Yellow	Grey
94.	Kz-66	KNP	E (LLS)	Undulate, pulvinate	Grey	Brown	Brown	Grey
95.	Kz-67	KNP	E (LLS)	Undulate, convex	Reddish brown	Brown	Brown	Variable
96.	Kz-69	KNP	E (LLS)	Curled, raised	Pink	Cream	ND	Variable
97.	Kz-70	KNP	E (LLS)	Erose, flat	Pink	Orange	ND	Variable
98.	Kz-72	KNP	E (LLS)	Undulate, convex	White	Cream	ND	White
99.	Kz-73	KNP	E (LLS)	Curled, umbonate	Grey	Reddish	ND	Grey
100.	Kz-74	KNP	E (LLS)	Erose, flat	Grey	Cream	ND	Grey
101.	Kz-75	KNP	E (LLS)	Entire, raised	White	Cream	ND	White
102.	Kz-76	KNP	E (LLS)	Undulate, convex	Grey	Grey	Brown grey	Grey
103.	Kz- 77	KNP	E (LLS)	Curled, pulvinate	Grey	Black	ND	Grey
104.	Kz-78	KNP	E (LLS)	Curled, umbonate	Brown	Reddish brown	Reddish brown	Variable
105.	Kz-79	KNP	E (LLS)	Curled, convex	White	Reddish	Brown	White
106.	Kz-80	KNP	E (LLS)	Curled, umbonate	Brown	Cream	Brown	Variable
107.	Kz-81	KNP	E (LLS)	Erose, convex	Purple grey	Purple	Purple	Grey

PWS: Pobitora Wildlife Sanctuary, Assam; KNP: Kaziranga National Park, Assam; GRS: Grass rhizosphere soil; LLS Leaf litter soil; TRS: Tree rhizosphere soil; SS: Sediment soil; ND: not detectable

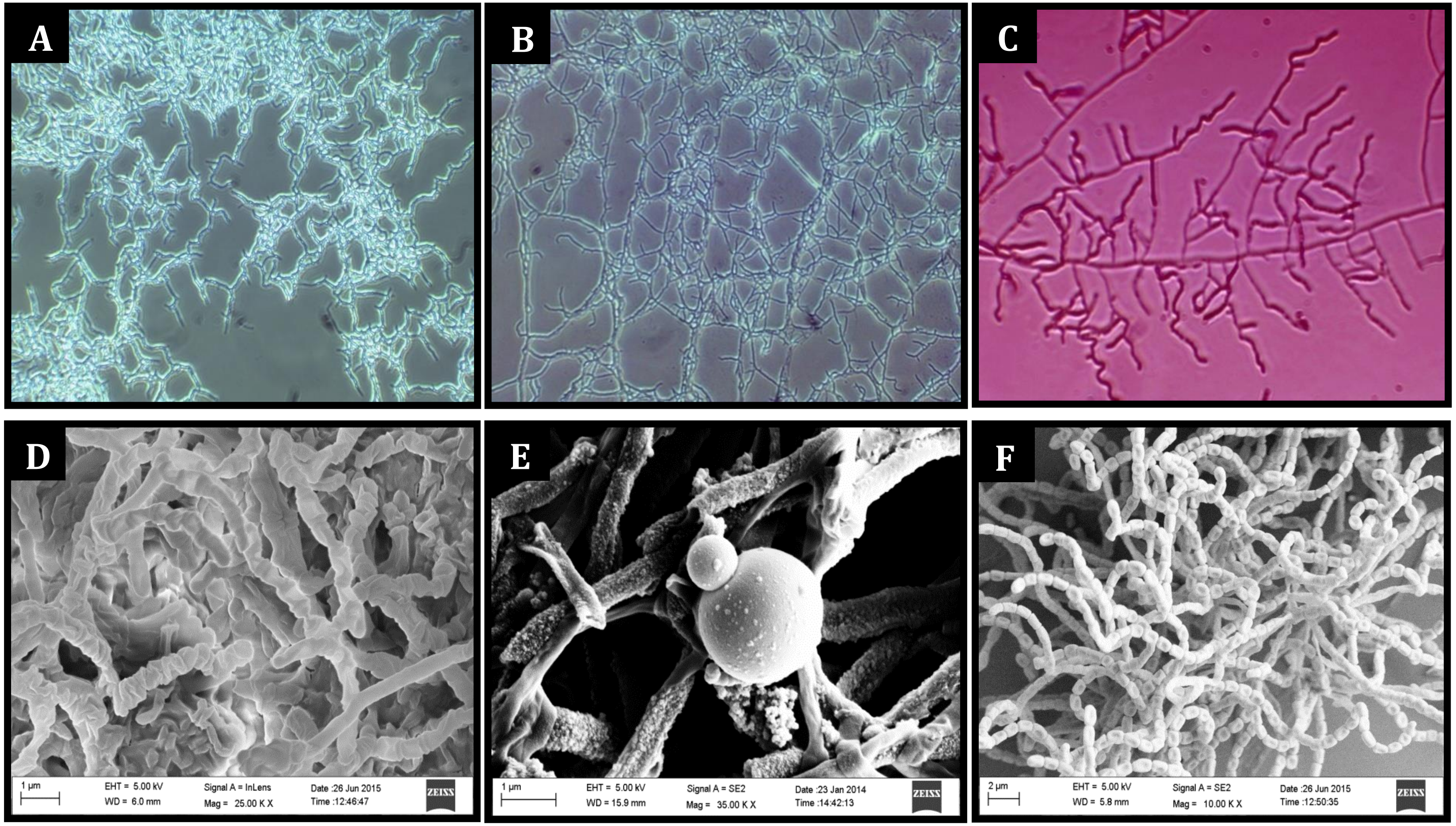

Supplementary Figure S1. Microscopic view of actinobacteria isolates showing aerial hyphae and spore chain morphology
Micro morphology using cover slip insertion method of (A) PB-31 (B) PB-48 (C) PB-52
Scanning electron micrograph view of (D) PB-52 (E) PB-65 (F) PB-76
[Figure (C) and (D) are reproduced from Sharma *et al*.[24]]

Supplementary Table S2. ***In vitro*** **secondary antimicrobial bioassay against test microorganisms with ethyl acetate crude extract recovered from actinobacteria cultures**

**Sl. no.**	**Isolate code**	**Isolation source**	***Zone of inhibition (mm)**									
			**Test microorganisms**									
			***S. aureus* MTCC 96**	***S. epidermidis* MTCC 435**	***B. subtilis* MTCC 441**	***M. luteus* MTCC 1538**	**MRSA ATCC [illegible]**	***E. coli* MTCC 40**	***S. marcescens* MTCC 97**	***K. pneumoniae* MTCC 3384**	***P. aeruginosa* MTCC 741**	***C. albicans* MTCC 227**
1.	PB-15	PWS	11^{a}±1	15^{c}±1.3	14^{bcd}±1.5	17^{g}±2	10^{ab}	12^{ab}±0.7	19^{efg}±1.5	13^{a}±1.5	22^{e}±1.4	15^{def}±1.1
2.	PB-21	PWS	11^{a}±1.2	14^{c}±1	11^{a}±1.1	11^{ab}±1.2	ND	17^{def}±1.2	16^{e}±1.5	14^{ab}±1.4	25^{f}±0.5	14^{cde}±1.1
3.	PB-28	PWS	16^{cd}±1.5	17^{d}±1	11^{a}±0.7	14^{def}±1.5	12^{cd}±1	20^{f}±1.2	16^{e}±1.2	13^{a}±1.2	30^{g}±1.1	16^{fg}±1.2
4.	PB-33	PWS	12^{a}±1	11^{a}±0.7	15^{cd}±0.8	15^{defg}±1.2	9^{a}±0.7	11^{ab}	ND	13^{a}±2	19^{d}±0.7	16^{efg}±0.7
5.	PB-43	PWS	13^{ab}±1.5	13^{abc}±1.8	17^{e}±1	13^{bcd}±1.2	12^{bcd}±0.5	10^{a}±1	9^{a}	43^{h}±1.2	16^{bc}±1.5	25^{h}±1.8
6.	PB-48	PWS	15^{bc}±1.5	14^{bc}±0.8	12^{ab}±1.3	14^{cde}±1	13^{de}±1.2	18^{ef}±0.2	10^{ab}±1.5	16^{bc}±1	15^{bc}±2	36^{j}±1
7.	PB-52	PWS	36^{h}±0.8	23^{f}±1.6	28^{j}±0.8	22^{h}±1.6	30^{i}±0.8	31^{h}±1.2	18^{d}±1.2	27^{e}±1.6	29^{h}±0.8	27^{h}±0.4
8.	PB-64	PWS	15^{bc}±1.5	12^{abc}±1.5	11^{a}±1.2	14^{cde}±1	15^{e}±1.6	14^{bcd}±1	10^{ab}	15^{abc}±1.1	15^{bc}±1.2	12^{abc}±2
9.	PB-65	PWS	18^{d}±1.8	17^{de}±1.1	18^{e}±1.7	17^{fg}±1.3	21^{gh}±1	31^{h}±0.5	11^{abc}±1.2	13^{a}±1.7	23^{ef}±1.5	28^{i}±1
10.	PB-68	PWS	11^{a}±2	11^{ab}±1.6	14^{bc}±1.7	10^{a}±1	11^{abc}±1	19^{ef}±2	12^{bcd}±1.2	28^{f}±1.5	24^{ef}±0.8	26^{h}±1.5
11.	PB-70	PWS	13^{ab}±1	ND	21^{f}±2	12^{abc}	12^{bcd}±2	13^{abc}±3	12^{bcd}±1.4	28^{f}±1.1	25^{f}±0.8	13^{bcd}±1.2
12.	PB-76	PWS	26^{f}±1.2	13^{abc}±1.2	22^{fg}±1.2	17^{g}±1	22^{h}±0.5	30^{h}±1.2	11^{abcd}±1.5	16^{bc}±1.2	22^{e}±1.5	15^{defg}±1
13.	Kz-13	KNP	11^{a}±0.7	13^{abc}±1.6	11^{a}±2	15^{defg}±1.2	15^{e}±2	17^{ef}±1.5	13^{cd}±2.2	20^{d}±0.7	14^{b}±1.2	11^{ab}±0.5

Sl. no.	Isolate code	Isolation source	*Zone of inhibition (mm)									
			Test microorganisms									
			S. aureus **MTCC 96**	***S. epidermidis*** **MTCC 435**	***B. subtilis*** **MTCC 441**	***M. luteus*** **MTCC 1538**	**MRSA ATCC 43300**	***E. coli*** **MTCC 40**	***S. marcescens*** **MTCC 97**	***K. pneumoniae*** **MTCC 3384**	***P. aeruginosa*** **MTCC 741**	***C. albicans*** **MTCC 227**
14.	Kz-24	KNP	$34^{h}\pm0.7$	ND	$25^{h}\pm2$	$36^{i}\pm1.5$	$40^{j}\pm0.8$	$41^{i}\pm1.5$	$19^{fg}\pm2.2$	$35^{g}\pm1.2$	28^{g}	$44^{k}\pm1$
15.	Kz-28	KNP	$22^{e}\pm1.2$	$19^{ef}\pm0.5$	$24^{gh}\pm0.7$	$16^{efg}\pm1.1$	$21^{gh}\pm0.5$	$25^{g}\pm1.5$	ND	$28^{f}\pm1$	$24^{f}\pm1.5$	$24^{h}\pm1.3$
16.	Kz-55	KNP	$29^{g}\pm1.5$	$31^{h}\pm2$	$27^{i}\pm0.5$	$15^{defg}\pm1.2$	$19^{g}\pm1.5$	$13^{ab}\pm1.1$	$17^{ef}\pm1$	$14^{ab}\pm1.4$	$11^{a}\pm1$	$16^{fg}\pm1$
17.	Kz-66	KNP	$23^{e}\pm1.7$	$24^{g}\pm2$	ND	$20^{h}\pm1$	$17^{f}\pm1.1$	$16^{cde}\pm1.7$	$17^{ef}\pm1.4$	$21^{d}\pm1.5$	$17^{c}\pm1.3$	10^{a}
18.	Kz-67	KNP	$16^{cd}\pm1.2$	$13^{abc}\pm1$	$17^{de}\pm1.4$	$21^{h}\pm1.7$	$21^{gh}\pm1.2$	$19^{ef}\pm4.6$	$17^{ef}\pm2.2$	ND	$16^{bc}\pm1.1$	$15^{defg}\pm1$
19.	Kz-74	KNP	17^{cd}	$20^{ef}\pm0.5$	$21^{f}\pm2$	$15^{defg}\pm1$	$19^{g}\pm1.5$	$20^{f}\pm1.7$	$20^{g}\pm0.5$	$17^{c}\pm1.3$	$17^{cd}\pm0.7$	$17^{g}\pm2.2$

*Zone of inhibition by disc diffusion method on GLM agar medium.
Size of the disc was 6 mm in diameter.
Zone of inhibition values are given as mean ± SD (n=3). Values having different superscripts (a-k) differ significantly ($P < 0.05$).
PWS: Pobitora Wildlife Sanctuary, Assam; KNP: Kaziranga National Park, Assam; ND: not detectable

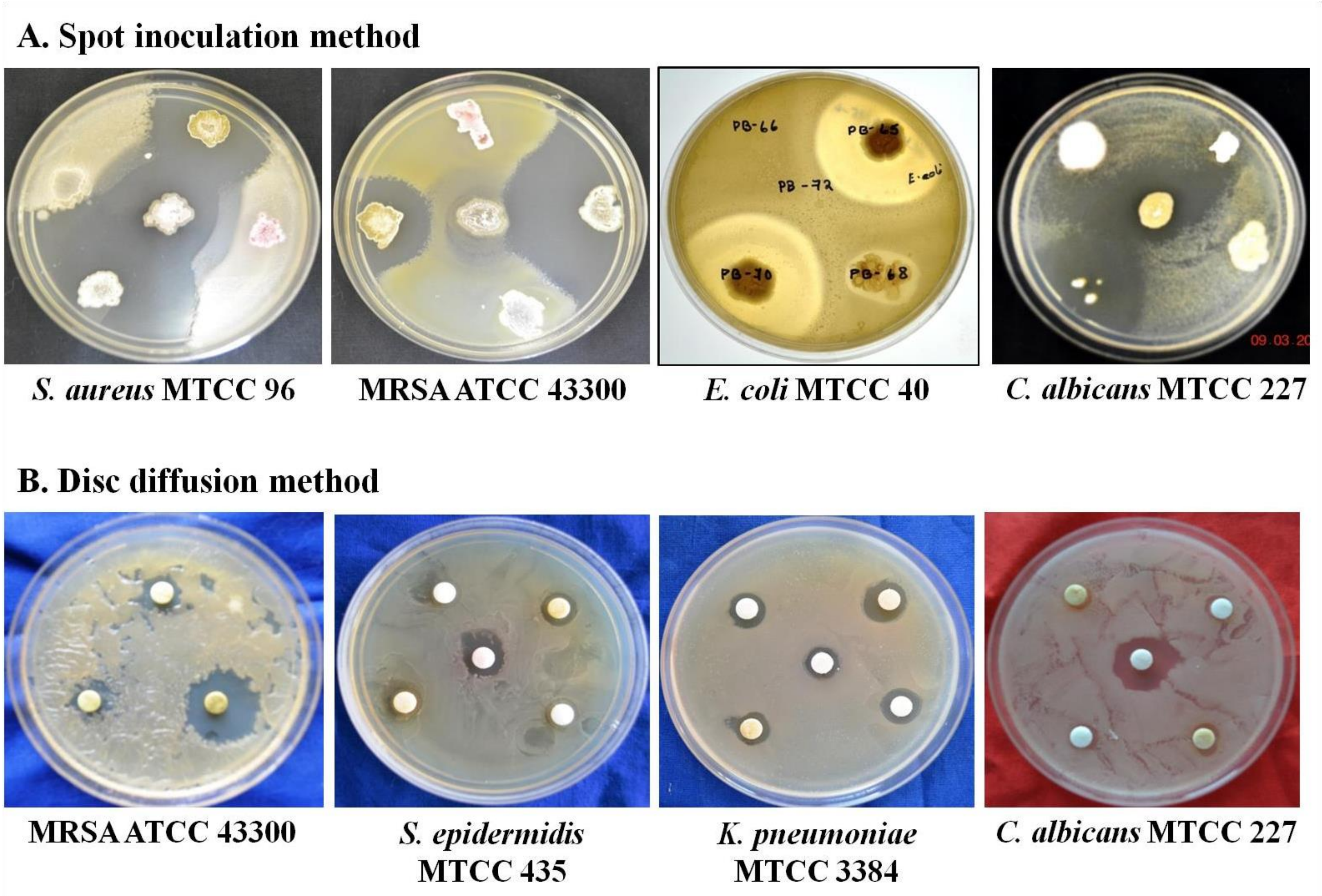

Supplementary Figure S2. *In vitro* antimicrobial activity of actinobacteria by (A) spot inoculation method and (B) disc diffusion method against test microorganisms.

Supplementary Table S3. Production of extracellular hydrolytic enzymes by actinobacteria isolated from Pobitora Wildlife Sanctuary and Kaziranga National Park of Assam, India

Sl. no.	Isolate code	Extracellular hydrolytic enzymes production				
		Amylase	Cellulase	Protease	Lipase	Esterase
1.	PB-9	+++	+++	-	-	++
2.	PB-10	-	+++	-	-	-
3.	PB-15	+++	++	-	-	+++
4.	PB-17	++	+++	++	-	++
5.	PB-19	+++	-	-	-	+++
6.	PB-20	+++	-	+	++	+++
7.	PB-21	+++	+	+++	+++	+++
8.	PB-22	+++	+++	++	++	+++
9.	PB-25	++	+++	++	-	++
10.	PB-26	+++	+++	+++	-	-
11.	PB-27	++	+++	++	+++	++
12.	PB-28	++	+	+++	-	-
13.	PB-31	-	-	-	-	-
14.	PB-32	-	++	+++	-	-
15.	PB-33	+++	+++	-	+++	+++
16.	PB-39	+++	-	+++	+++	+++
17.	PB-43	+++	+++	+++	+++	+++
18.	PB-46	++	+++	-	++	++
19.	PB-47	-	-	+	-	++
20.	PB-48	-	+++	+++	+++	-
21.	PB-50	+++	+++	+++	+++	+++
22.	PB-51	+++	+++	-	++	+++
23.	PB-52	+++	-	-	++	-
24.	PB-54	+++	+++	+++	+++	-
25.	PB-55	+++	+++	+++	++	-
26.	PB-56	++	-	-	+++	++
27.	PB-64	-	+++	+++	++	++
28.	PB-65	+++	-	++	-	-
29.	PB-66	++	+++	-	++	+++
30.	PB-68	-	++	++	++	-
31.	PB-70	+++	+++	+	++	++
32.	PB-75	+++	++	++	+	+
33.	PB-76	+++	-	+++	+++	+++
34.	PB-79	+++	+++	++	+++	+++
35.	PB-81	+++	++	+++	+++	++
36.	PB-82	+++	++	+++	+++	++
37.	PB-83	+++	-	+++	+++	-
38.	PB-85	+++	+	+++	++	++
39.	PB-86	+++	+++	+++	+	-
40.	Kz-2	+++	-	-	++	-
41.	Kz-10	+++	+++	+++	+	+
42.	Kz-11	+++	-	+++	+++	+++
43.	Kz-12	+++	+	+	+	+
44.	Kz-13	-	+++	-	-	++

Sl. no.	Isolate code	Extracellular hydrolytic enzymes production				
		Amylase	Cellulase	Protease	Lipase	Esterase
45.	Kz-14	+++	+++	-	+++	+
46.	Kz-18	+++	+++	-	+++	+++
47.	Kz-21	+++	++	-	+++	+++
48.	Kz-23	++	-	+	+	++
49.	Kz-24	+++	+++	-	++	+
50.	Kz-27	+++	+++	-	-	+
51.	Kz-28	+++	+++	+++	+++	+++
52.	Kz-29	+++	-	+++	+++	+++
53.	Kz-31	-	+++	-	-	+
54.	Kz-32	+++	+	+++	++	++
55.	Kz-36	+++	-	++	+++	+++
56.	Kz-38	-	++	-	-	++
57.	Kz-41	+++	+++	++	+++	+++
58.	Kz-42	-	++	-	-	-
59.	Kz-44	+++	+	+	+++	+++
60.	Kz-47	+++	+++	++	+++	+++
61.	Kz-49	+++	-	+	++	+
62.	Kz-52	+++	+++	+	++	+++
63.	Kz-55	+++	+++	-	++	++
64.	Kz-56	+++	-	++	+	-
65.	Kz-58	+++	+++	++	-	+
66.	Kz-61	-	+	+	+	-
67.	Kz-62	-	-	+++	+++	+++
68.	Kz-66	+++	+++	+++	+++	+++
69.	Kz-67	+++	-	+++	+++	+++
70.	Kz-72	-	+++	-	+	-
71.	Kz-73	+++	++	+++	+++	-
72.	Kz-74	+++	+++	+++	++	+++
73.	Kz-75	++	-	-	+	++
74.	Kz-76	+++	+++	+++	++	+++
75.	Kz-78	+++	-	+++	+++	+++
76.	Kz-79	+++	+++	+++	++	+++
77.	Kz-80	+++	+++	+++	++	+++

+++ good production (≤ 18 mm diameter of zone of clearance or precipitate); ++ moderate production (14-17 mm); + poor production (≥ 13 mm); ND: not detectable; PWS: Pobitora Wildlife Sanctuary, Assam; KNP: Kaziranga National Park, Assam. The average size of colonies of the actinobacteria isolates were (7±2) mm in diameter after 5-10 days of incubation at 28°C.

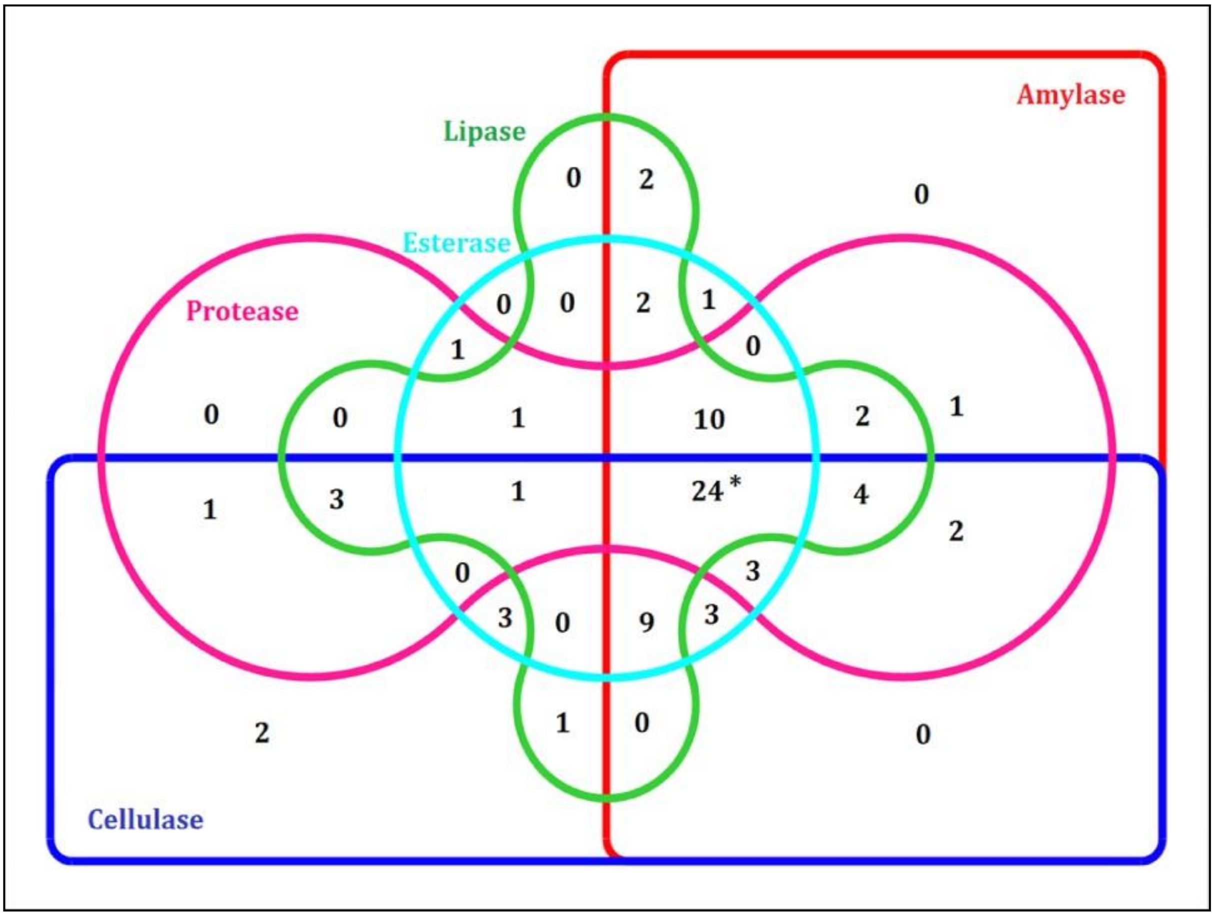

Supplementary Figure S3. Venn diagram representation of production of extracellular hydrolytic enzymes i.e. amylase, cellulase, protease, lipase and esterase by the actinobacteria isolates using VENNTURE software (24* isolates produced all the five enzymes tested)

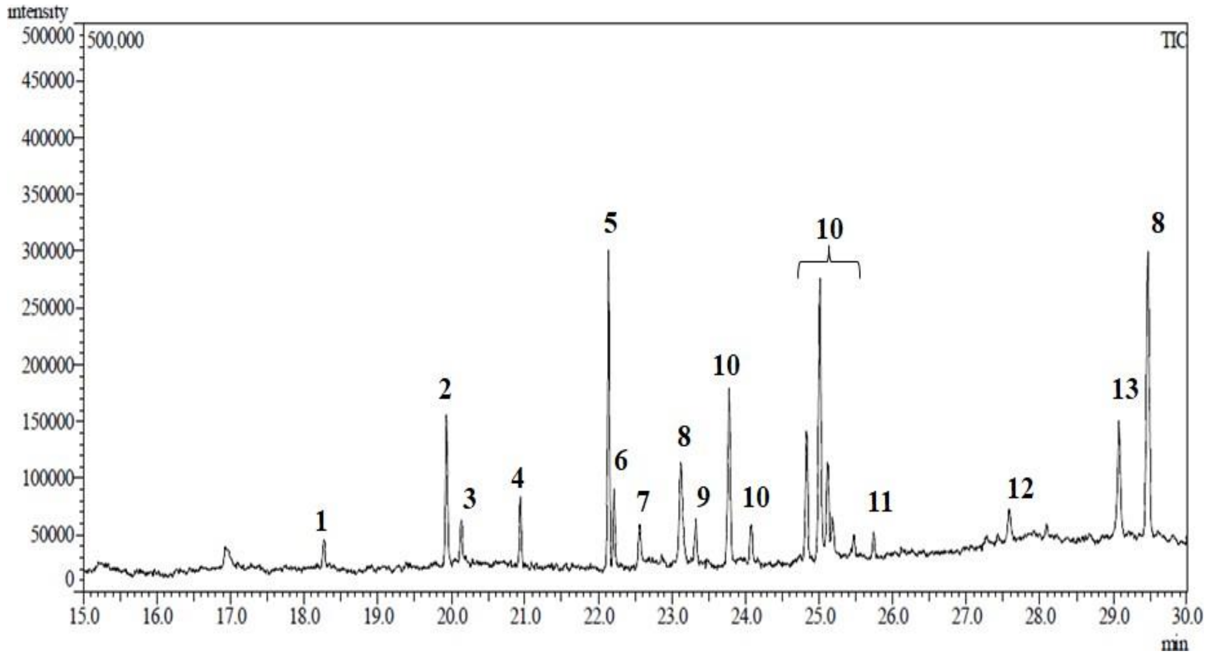

Supplementary Figure S4. GC-MS profile of EA-Kz-24 extracted from Kz-24.
The numbers above the peaks in the chromatogram indicates the 13 compounds identified in EA-Kz-24.

1. (Z)-3-Tridecene; **2.** 3,5-bis(1,1-dimethylethyl)-phenol; **3.** Benzoic acid,4-ethoxy-ethyl ester; **4.** (Z)-3-Tetradecene; **5.** Dodecyl acrylate; **6.** Propanoic acid, decyl ester; **7.** N-acetyl-3-methyl-1,4-diazabicyclo[4.3.0]nonan-2,5-dione; **8.** Hexahydro-pyrrolo[1,2-a]pyrazine-1,4-dione; **9.** (E)-9-Octadecene; **10.** Hexahydro-3-(2-methylpropyl)-pyrrolo[1,2-a]pyrazine-1,4-dione; **11.** Propanoic acid,3-mercapto-dodecyl ester; **12.** 3-(phenylmethyl)-2,5-piperazinedione; **13.** Hexahydro-3-(phenylmethyl)-pyrrolo[1,2-a]pyrazine-1,4-dione
